# Construction Immune Related Feed-Forward Loop Network Reveals Angiotensin II Receptor Blocker as Potential Neuroprotective Drug for Ischemic Stroke

**DOI:** 10.3389/fgene.2022.811571

**Published:** 2022-03-28

**Authors:** Chunrui Bo, Yuze Cao, Shuang Li, Huixue Zhang, Xiaoyu Lu, Xiaotong Kong, Shuai Zhang, Hongyu Gao, Jianjian Wang, Lihua Wang

**Affiliations:** ^1^ Department of Neurology, The Second Affiliated Hospital, Harbin Medical University, Harbin, China; ^2^ Department of Neurology, Peking Union Medical College Hospital, Chinese Academy of Medical Sciences, Beijing, China

**Keywords:** ischemic stroke, immune, feed-forward loops, neuroprotection, angiotensin II receptor blockers

## Abstract

Ischemic stroke (IS) accounts for the leading cause of disability and mortality in China. Increasing researchers are studying the effects of neuroprotective agents on IS. However, the molecular mechanisms of feed-forward loops (FFLs) associated with neuroprotection in the pathogenesis of IS need to be further studied. A protein-protein interaction (PPI) network of IS immune genes was constructed to decipher the characters and excavate 3 hub genes (PI3K, IL6, and TNF) of immunity. Then, we identified two hub clusters of IS immune genes, and the cytokine-cytokine receptor interaction pathway was discovered on the pathway enrichment results of both clusters. Combined with GO enrichment analysis, the cytokines participate in the inflammatory response in the extracellular space of IS patients. Next, a transcription factor (TF)–miRNA–immune gene network (TMIGN) was established by extracting four regulatory pairs (TF–miRNA, TF–gene, miRNA–gene, and miRNA–TF). Then, we detected 3-node regulatory motif types in the TMIGN network. According to the criteria we set for defining 3-node motifs, the motif with the highest Z-score (3-node composite FFL) was picked as the statistically evident motif, which was merged to construct an immune-associated composite FFL motif-specific sub-network (IA-CFMSN), which contained 21 3-node FFLs composed of 13 miRNAs, 4 TFs, 9 immune genes, and 1 TF& immune gene, among which TP53 and VEGFA were prominent TF and immune gene, respectively. In addition, the immune genes in IA-CFMSN were used for identifying associated pathways and drugs to further clarify the immune regulation mechanism and neuroprotection after IS. As a result, 5 immune genes targeted by 20 drugs were identified and the Angiotensin II Receptor Blockers (ARBs) target AGTR1 was found to be a neuroprotective drug for IS. In the present study, the construction of IA-CFMSN provides IS immune-associated FFLs for further experimental studies, providing new prospects for the discovery of new biomarkers and potential drugs for IS.

## Introduction

Stroke is the leading cause of disability and mortality in China (Global, 2018), of which ischemic stroke (IS) or transient ischemic attack (TIA) make up about 70% of all strokes (Wang et al., 2011). The treatment of acute ischemic stroke (AIS) first focuses on rapid revascularization, but this requires a narrow time window within 4.5 h after stroke onset, and merely about 2–5% of stroke patients are eligible for recombinant tissue plasminogen activator (rt-PA) (Rabinstein, 2020). Therefore, a majority of patients may benefit from an alternative treatment. The pathophysiology of ischemic stroke is a complex consequence of multiple mechanisms, including oxidative stress, blood-brain barrier disruption, inflammation, and apoptosis (Rodrigo et al., 2013; Chamorro et al., 2016; Jiang et al., 2018). So far, the immunotherapy of IS has provided preliminary results from the perspective of clinical studies (Fu et al., 2014; Zhu et al., 2015; Zhang et al., 2017). In terms of molecular mechanism, there has been a tremendous increase in the studies of IS from protein-coding genes to non-coding RNAs. Moreover, possible changes in miRNAs generated by immunomodulatory treatments for IS may also play an important role (Martinez and Peplow, 2017). However, the molecular mechanisms and drugs for neuroprotection and immunomodulation behind the pathogenesis of IS are still warranted.

In our previous study (Bo et al., 2021), we mentioned two types of pivotal gene regulators: transcription factors (TFs) and miRNAs, both sharing a common regulatory logic in the co-regulation of target genes (Hobert, 2008). Besides, gene regulators (miRNA and TF) and their common target gene can make up a feed-forward loop (FFL), a motif that consists of two regulators and their common target gene (Mangan and Alon, 2003).

Substantial evidence has demonstrated that miRNAs and TFs concur to explain the neuroprotective mechanism of IS. The upregulation of a variety of neuroprotective genes has been found to be associated with some specific miRNAs such as miR-762, -1892, -200a, -145, thereby demonstrating their potential neuroprotective effects (Martinez and Peplow, 2017). MiR-124, -711, -145 are predicted to mediate the activation phenotype of microglia/macrophage to M2-like and anti-inflammatory pathways (Martinez and Peplow, 2017). Recent evidence also demonstrated that activating transcription factor 6 (ATF6) can mitigate adverse outcomes associated with ischemia/reperfusion injury (Glembotski et al., 2019). A Notch intracellular domain (NICD)/p53 interaction is involved in transcriptional regulation of genes downstream of p53 and NICD, thereby alleviating the severity of stroke (Balaganapathy et al., 2018). In addition, a five-node FFL network was constructed to describe the interactions of TFs, miRNAs, and genes in IS, and STAT and NFKB were identified as the main regulatory TFs of innate inflammatory and neuronal survival mechanisms to explain the possible transcriptional mechanism of IS (Nampoothiri et al., 2018). The FFLs might be the explanation for the fact of multifactorial nature in IS pathology. However, the immune-associated FFLs in IS have not been studied. Therefore, immune-associated FFLs could be used for deciphering the underlying post-transcriptional mechanisms and providing new clues for neuroprotection of IS.

In the present study, a protein-protein interaction (PPI) network of IS immune genes was constructed to excavate the hub genes of immunity. Then, we identified two hub clusters of IS immune genes, and discovered the importance of the Cytokine-cytokine receptor interaction pathway through pathway enrichment analysis. Combined with GO enrichment analysis, these results suggested that the cytokines participate in the inflammatory response in the extracellular space in IS. Next, a transcription factor (TF)–miRNA–immune gene network (TMIGN) was established, where 3-node regulatory motif types were detected. As a result, the 3-node composite FFL was picked as the statistically evident motif, which was merged to construct an immune-associated composite FFL motif-specific sub-network (IA-CFMSN). In addition, the immune genes in IA-CFMSN were used to identify associated pathways and drugs. Interestingly, we filtered ARBs through IA-CFMSN as potential drugs to provide new insight into the neuroprotective mechanisms for IS. In conclusion, the present study highlighted the effect of immune-associated FFLs in IS, which revealed their possibility as neuroprotective biomarkers in IS.

## Methods

### Acquirement of IS-Related Immune Genes, TFs, and miRNAs

IS genes were obtained through the GeneCards database (Stelzer et al., 2016) (https://www.genecards.org/) by searching with keyword “ischemic stroke” and then we selected genes with a relevance score>10 and protein-coding genes as IS-related genes (Supplementary Table S1). Next, IS immune-related genes (Supplementary Table S2) were acquired by intersecting IS-related genes and immune genes from the ImmPort database (Bhattacharya et al., 2018) (https://www.immport.org/home).

MicroRNAs involved in IS were obtained in two ways: We first collected miRNAs which were verified by low–output experiments and restricted to “*Homo sapiens*” through the Nervous System disease NcRNAome Atlas database (NSDNA) (Wang et al., 2017) (http://www.bio-bigdata.net/nsdna/) before 2017. Then we searched the PubMed database to screen candidate IS-associated miRNAs before May 2021. Then, IS-associated miRNAs were screened in patients with ischemic stroke, whose miRNA expression levels are significantly different from those in the control group, and are confirmed by reliable low-throughput experiments, such as real-time fluorescent quantitative PCR. The names of miRNAs were finally unified by the miRBase database (Kozomara et al., 2019) (http://www.mirbase.org/). All miRNAs were listed in Supplementary Table S3.

The TFs were acquired from the union of three databases: transcriptional regulatory element database (TRED) (Jiang et al., 2007) (http://rulai.cshl.edu/TRED), ChIPBase v2.0 (Zhou et al., 2017) (http://rna.sysu.edu.cn/chipbase/), and Transcriptional Regulatory Relationships Unraveled by Sentence-based Text mining (TRRUST) (Han et al., 2018) (http://www.grnpedia.org/trrust/). In addition, TFs presented in the collected IS gene list were selected as IS risk TFs (Supplementary Table S4).

### Establishment of the PPI Network, Hub Gene Identification and Module Analysis

The PPI network of IS immune genes with a combined score of interactions >0.9 was identified using the STRING database (http://string-db.org/) (Szklarczyk et al., 2017). Subsequently, the PPI network was visualized by Cytoscape software (v 3.7.0). Nodes with a connective degree >30 were defined as the hub genes in this PPI network by the CentiScaPe v2.0 plugin for Cytoscape (Scardoni et al., 2009). The Molecular Complex Detection (MCODE) plugin (Bader and Hogue, 2003) was used to detect significant modules with MCODE scores≥5, node score cutoff = 0.2, Max depth = 100, degree cutoff = 2, and k-core = 3.

### Functional Enrichment Analysis

Functional annotation tools in DAVID, including KEGG pathway enrichment analysis and Gene Ontology (GO) annotation were applied to identify the risk pathways and GO terms in which IS immune genes were enriched. It was considered significantly enriched if it displayed a *p*-value <0.05.

### Generation of Regulatory Interaction Pairs Between miRNAs, Genes, and TFs

miRNA-gene pairs were extracted from miRTarbase (Hsu et al., 2014) (http://mirtarbase.mbc.nctu.edu.tw/php/index.php). miRNA-gene pairs were picked when they showed strong evidence of interaction and were in compliance with IS gene list. TFs were considered as genes when finding miRNA–TF interactions, which were also obtained from miRTarbase (Hsu et al., 2014) using the same procedure as miRNA-gene pairs. In addition, the TF-miRNA and TF-gene pairs were acquired from TransmiR v2.0 (Wang et al., 2010) (http://www.cuilab.cn/transmir) and TRRUST (Han et al., 2018) (http://www.grnpedia.org/trrust/), respectively.

### Detection of 3 Node Network Motifs and Definition of 3-Node Composite FFL

Four types of regulation were incorporated to construct a TF-miRNA-immune gene network. These regulatory relationships (TF→miRNA, TF→Gene, miRNA→Gene, and miRNA→TF) can form different types of 3-node motifs. Therefore, fast network motif detection (FANMOD) software was used to detect network motif types of size-3 in the original network (Wernicke and Rasche, 2006). The method of generating motifs by FANMOD software can be found in our previous study (Bo et al., 2021). Finally, 3-node motif types with Z-score >2.0 and *p*-value < 0.05 were considered to be significant in the dumping results of multiple types of motifs (Harari and Liao, 2010).

Following our previous study (Bo et al., 2021), we defined a qualified motif as a composite FFL. Therefore, a 3-node motif, as well as a 3-node composite FFL, is required to contain 1 TF, one miRNA, one common target gene, and all four types of regulatory relationships listed above.

### Generation of Motif Specific Sub-Network and Topological Features of Networks

The 3-node composite FFLs filtered following the above criteria were further selected to generate the sub-network. The motif-specific sub-network was constructed by merging motifs of the same kind. Therefore, an immune-associated composite FFL motif-specific sub-network (IA-CFMSN) was constructed. All the networks were built using the Cytoscape software (v 3.7.0). The topological features, such as the degrees, connectivity, topological coefficient, and clustering coefficient of nodes were analyzed to reveal the characteristics of the networks.

### Drugs and Drug Targets Data

The data of drugs and their target genes were downloaded from the DrugBank database (Wishart et al., 2018) (https://www.drugbank.ca/) (version 5.1.2). The species was limited to “*Homo sapiens*”.

### Cumulative Hypergeometric Distribution

Cumulative hypergeometric distribution was used to identify the significant correlations between miRNAs in IA-CFMSN and drugs. The formula was as follows:

$$P=F\left(\text{x|}M,K,N\right)=\overset{x}{\underset{i-0}{\sum }}\frac{\left(\begin{array}{c} K\\ i \end{array}\right)\left(\begin{array}{c} M-K\\ N-i \end{array}\right)}{\left(\begin{array}{c} M\\ N \end{array}\right)}$$ (1)

Here, M denoted the total number of the whole human genome, K denoted the number of target genes in miRNAs, the N denoted the number of target genes in drugs, and x represented the number of overlapping genes between miRNAs and drugs. We believed that it had statistical significance if *p*-value was less than 0.05.

## Results

### Construction of the PPI Network of IS Immune Genes and Module Analysis

**FIGURE 1:**
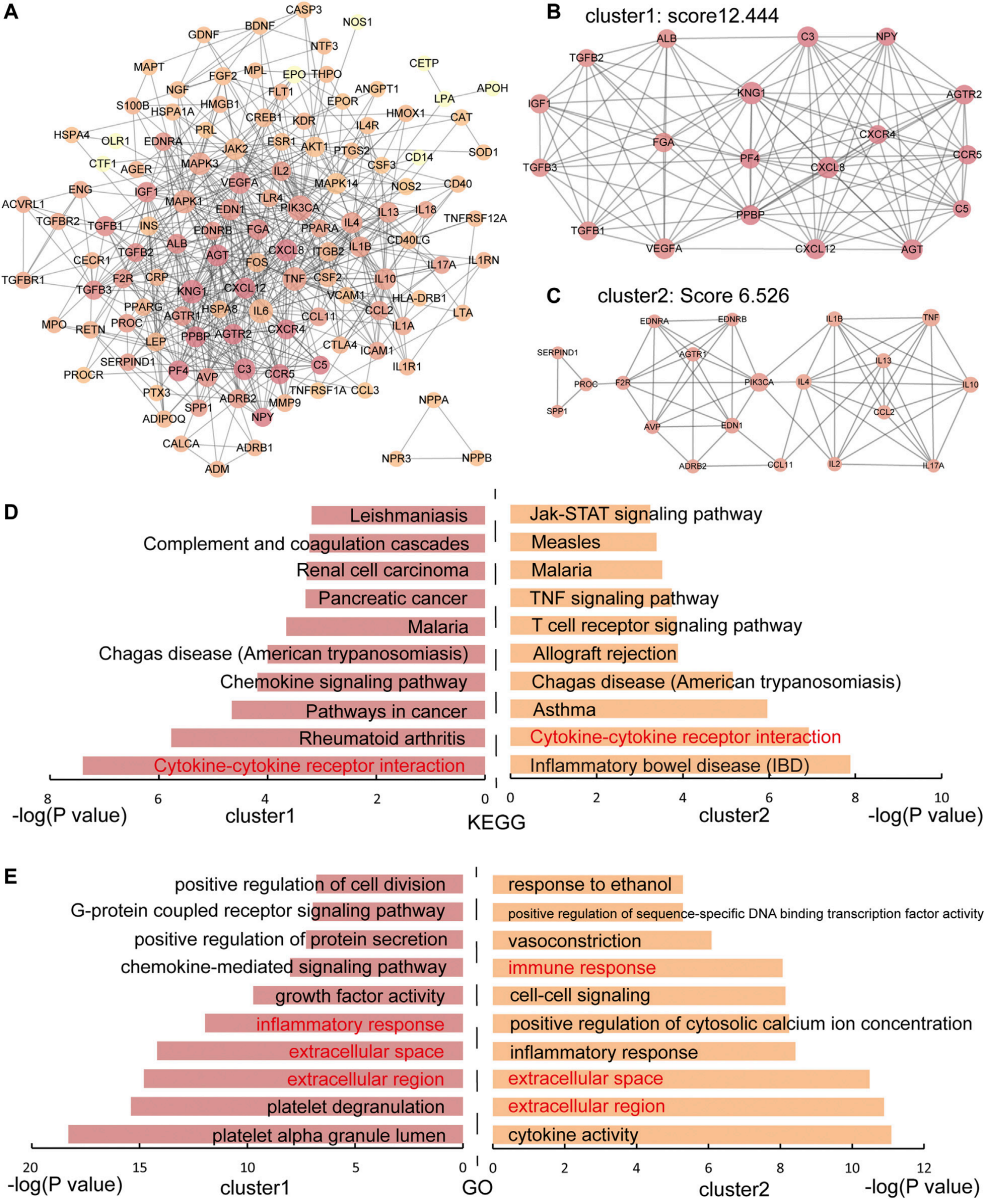
The PPI network and functional enrichment analysis of IS immune genes. **(A)** The PPI network of IS immune genes. The darker the node, the greater the degree. **(B-C)** Two significant modules in the PPI network with MCODE scores≥5. **(D)** KEGG enrichment analysis of immune genes in cluster 1 and cluster 2. **(E)** GO enrichment analysis of immune genes in cluster 1 and cluster 2. Shared pathways and processes between two clusters are shown in red.

The PPI network of IS genes was constructed and the distribution of immune genes was marked in Supplementary Figure S1. Then, to explore the interaction among the 136 IS immune genes, a PPI network of immune genes was constructed (Figure 1A). The PPI network of IS immune genes contained 122 nodes and 628 edges. Two significant clusters were identified using the MCODE plug-in. Cluster 1 contained 19 nodes and 112 edges, which exhibited the highest score of 12.444 (Figure 1B). Cluster 2 containing 20 nodes and 62 edges, also possessed a strong connection with a score of 6.526 (Figure 1C). Furthermore, the hub genes with a connectivity degree >30 were identified using the CentiScaPe v2.0 plugin. Therefore, the most significant 4 genes were PIK3CA, IL6, TNF, and KNG1, which means these molecules may function as important immune regulators in the inflammatory response following ischemic stroke.

### Enrichment Analysis of IS Immune Genes

**FIGURE 2:**
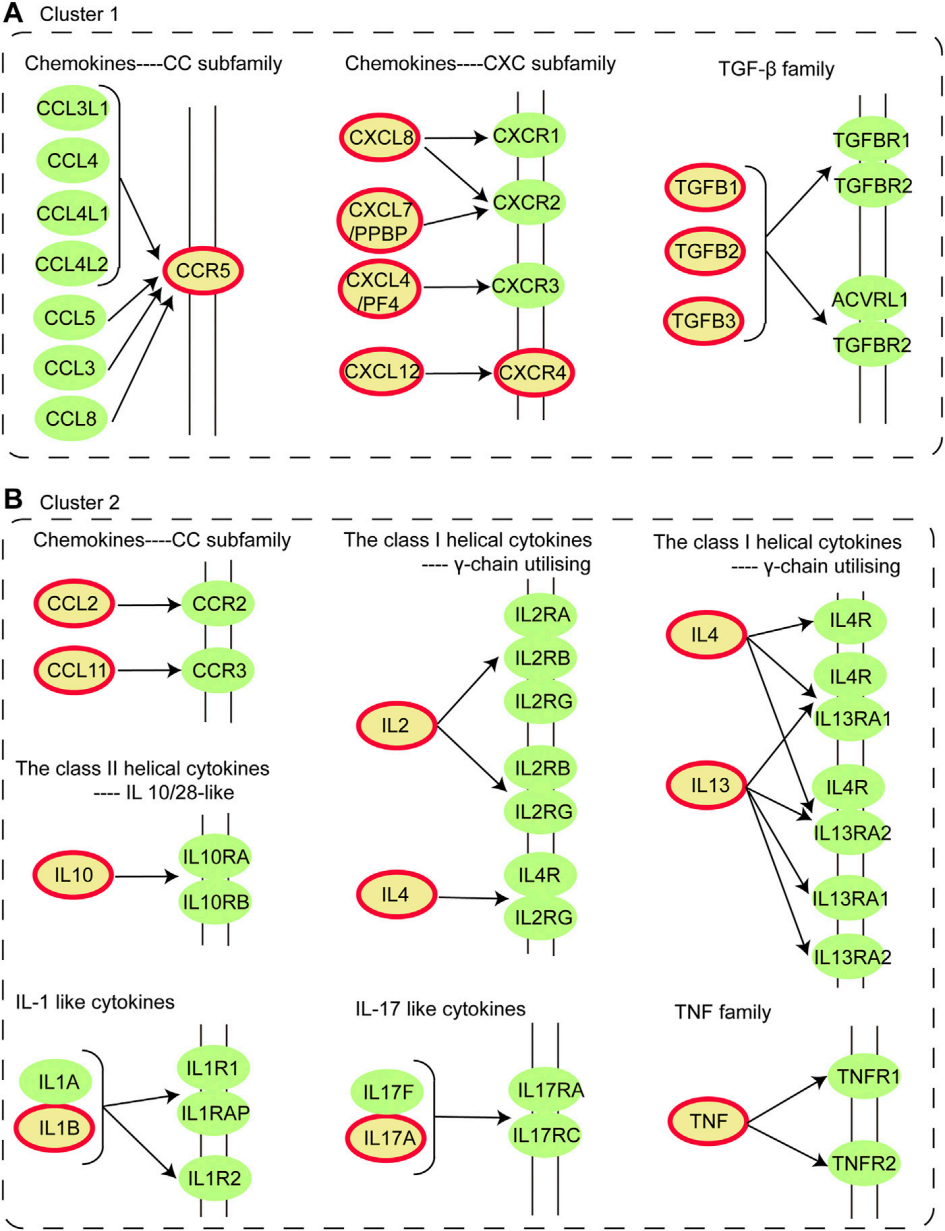
Dissection of Cytokine-cytokine receptor interaction pathway. **(A)** Distribution of immune genes in Cluster 1 in Cytokine-cytokine receptor interaction pathway. **(B)** Distribution of immune genes in Cluster 2 in Cytokine-cytokine receptor interaction pathway. The nodes colored in yellow with a red circle are immune genes identified in Cluster 1 and 2.

o better understand the characteristic of IS immune genes, we applied the functional enrichment analysis of genes in cluster 1 and cluster 2 respectively and the KEGG and GO enrichment results were shown in Figures 1D,E respectively. We found that the Cytokine-cytokine receptor interaction pathway was discovered on the pathway enrichment results of both clusters. Thus, we dissected the pathway of Cytokine-cytokine receptor interaction, nine immune genes in Cluster 1 and 10 immune genes in Cluster 2 participated in cytokine-cytokine receptor interaction (Figure 2). The immune genes in IS were mainly focused on chemokines (CC and CXC subfamily), interleukins, TGF-β family, and TNF family, which means these cytokines may play crucial roles in regulating immune response following ischemic stroke. Besides, the GO terms enriched by both clusters were “extracellular region”, “extracellular space” and “inflammatory response”, which further proved the above assumptions that the cytokines participate in the inflammatory response in the extracellular space in IS patients.

### Interaction Pairs Among IS-Related miRNAs, Immune Genes and TFs

We finally collected 136 IS immune genes, 117 miRNAs, and 20 TFs, among which, 6 TFs(CREB1, ESR1, FOS, PPARA, PPARG, and VDR)were TF& immune genes, which are TFs and immune-related genes simultaneously. Among 240 miRNA-gene pairs, 70 IS immune genes were found to be validated targets for 74 miRNAs. Hsa-miR-146a-5p has the largest number of target genes, and VEGFA is regulated by the largest number of miRNAs. Of 94 miRNA-TF pairs, hsa-miR-27a-3p is the miRNA that regulates the most TFs, and as a TF, TP53 can receive the regulation of the largest number of miRNAs. In addition, among 131 TF-gene pairs, as a TF, HIF1A was identified to regulate the largest number of IS immune genes, while VEGFA received the most TF regulation. Among 167 regulatory pairs of TF-miRNA pairs, it was found that the transcription factor TP53 regulated the most number of miRNAs and hsa-miR-145-5p was the top miRNA regulated by TFs. All the association information of interaction pairs is summarized in Table 1.

**TABLE 1:** Summary of four types of regulatory relationships among IS-related miRNAs, immune genes, and TFs.

Relationship	Number of pairs	Number of miRNAs	Number of genes	Number of TFs
miRNA-gene^a^	240	74	70	—
miRNA-TF^b^	94	48	—	16
TF-miRNA^c^	167	76	—	15
TF-gene^d^	131	-	62	18

a miRNA, represses the gene expression.

b miRNA, represses the TF, expression.

c TF, regulates the miRNA, expression.

d TF, regulates the gene expression.

### Construction of TF–miRNA–Immune Gene Network for IS

**FIGURE 3:**
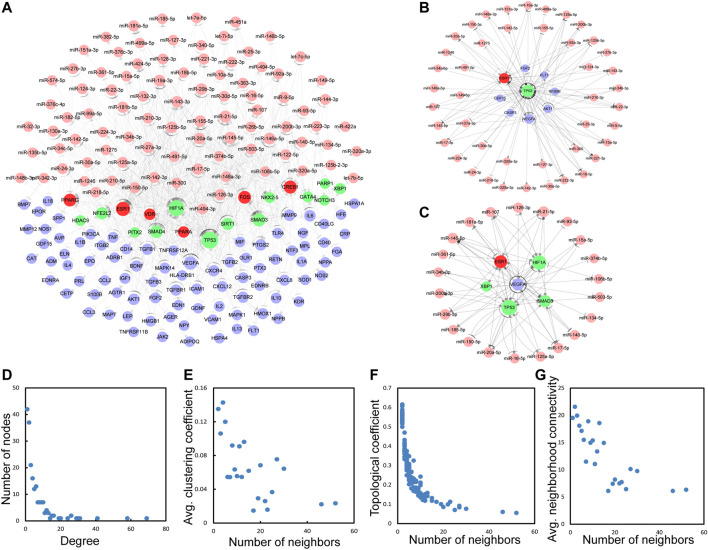
The basic characteristics of the network with four types of regulatory pairs (miRNA-gene, miRNA-TF, TF-miRNA, TF-gene). **(A)** A global of the network with four regulatory pairs. TFs, miRNAs, immune genes and TF&immune genes are colored green, pink, blue and red, respectively. **(B)** A sub-network with TP53 as the central node. **(C)** A sub-network with VEGFA as the central node. **(D–G)** The basic features of the network include degrees, clustering coefficient, topological coefficient and neighborhood connectivity of the network.

Next, a global view of the TF–miRNA–immune gene network (TMIGN) (Figure 3A) for IS was constructed by merging the four regulatory pairs (miRNA–TF, TF–miRNA, miRNA–gene, and TF–gene) identified above. TMIGN was composed of 195 nodes (13 TFs, 6 TF & immune genes, 90 miRNAs and 86 immune genes) and 594 edges. In TMIGN, TP53 had the largest degrees in the transcription factors, and VEGFA had the largest degree in immune genes, so we generate the sub-networks with TP53 and VEGFA as the central node and their first neighbors (Figures 3B,C), suggesting their important roles in maintaining the overall connectivity of the network (Yu et al., 2004). The topological features of TMIGN were also analyzed, including degrees, clustering coefficients, topological coefficients and neighborhood connectivity (Figures 3D–G). The degree distribution of TMIGN presented a power law distribution f(x) = 61.753x^(−1.158) with an *R* ^2^ of 0.878, indicating that TMIGN presented a scale-free distribution and a small-world phenomenon (Amaral et al., 2000).

### Detection of 3-Node Network Motifs and 3-Node Composite FFL

**FIGURE 4:**
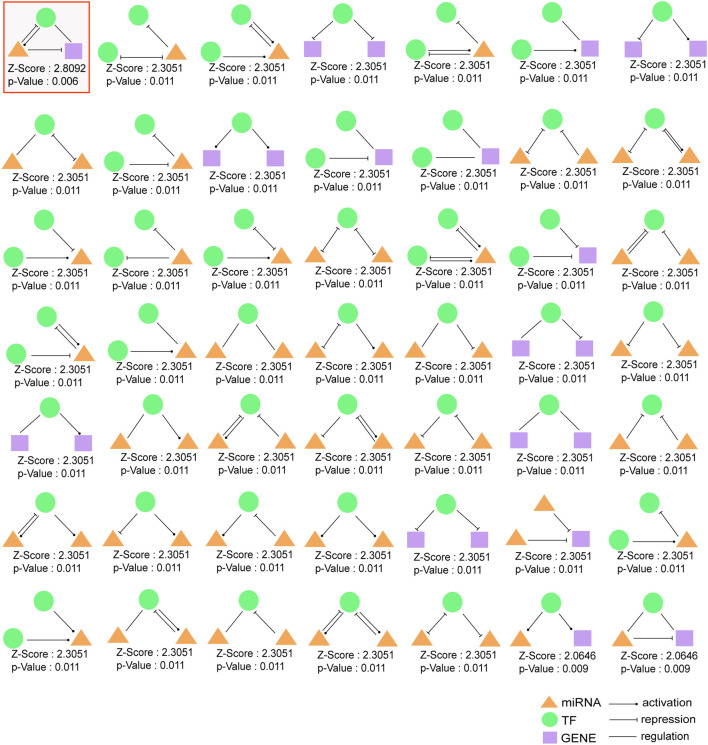
49 different types of 3-node regulatory motifs. The motifs are composed of miRNAs, TFs, and immune genes and their Z-scores and *p*-values are presented. Orange triangle represents miRNA, green round represents transcription factor (TF) and purple square represents immune gene. An arrow ending in a circle represents the activation relationship, an arrow ending in a “T” represents the repression relationship, while, a straight line without an arrow represents regulatory relationship. Motif surrounded by red squares (3-node composite feed-forward loop (FFL)), regarded as significant motif, and was merged to form 3-node regulatory sub-network.

Three-node regulatory motifs were detected in TMIGN, as a result, 49 different types of 3-node network motifs (Figure 4) were identified using the FANMOD tool. Here, we intended to investigate motifs with Z-score higher than 2, *p*-value less than 0.05, and met the criteria we set for a qualified 3-node motif, as well as a 3-node composite FFL. Therefore, as compared with other motif types, only one motif, as well as the 3-node composite FFL (surrounded by a red square in Figure 4) met the above requirement, and was selected as the statistically significant motif (Z-score: 2.8092, *p*-value: 0.006), which was merged to form the 3-node composite FFL regulatory sub-network.

### Generation of Immune-Associated Composite FFL Motif-Specific Sub-Network

**FIGURE 5:**
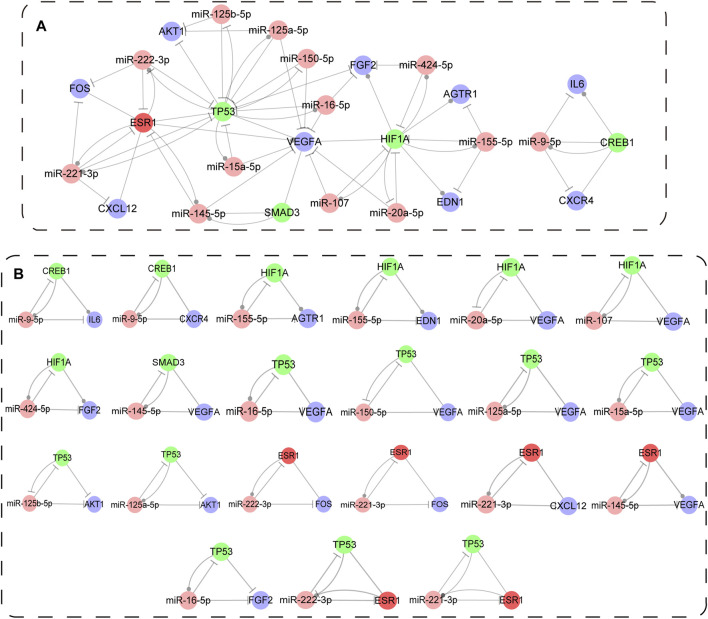
Immune associated composite FFL motif-specific sub-network (IA-CFMSN). **(A)** A global view of immune associated composite FFL motif-specific sub-network (IA-CFMSN). Pink represents miRNAs, TFs, genes and TF&gene were colored in pink, green, blue and red, respectively. A line ending in a circle represents the active relationship, while, a line ending in a “T” represents the repression relationship. **(B)** All 3-node composite FFLs extracted from IA-CFMSN.

The regulatory sub-network, as well as immune-associated composite FFL motif-specific sub-network (IA-CFMSN) was shown in Figure 5A. IA-CFMSN was composed of 13 miRNAs (miR-9-5p, miR-145-5p, miR-221-3p, miR-222-3p, miR-107, miR-155-5p, miR-20a-5p, miR-424-5p, miR-125a-5p, miR-125b-5p, miR-150-5p, miR-15a-5p and miR-16-5p), 4 TFs (CREB1, HIF1A, SMAD3 and TP53), 9 immune genes (CXCR4, IL6, CXCL12, FOS, VEGFA, AGTR1, EDN1, FGF2 and AKT1) and 1 TF& immune gene (ESR1). Then, all of the 3-node composite FFL was extracted from IA-CFMSN, shown in Figure 5B. The topological features of IA-CFMSN were also analyzed, including degrees, clustering coefficients, topological coefficients, and neighborhood connectivity (Supplementary Figure S2). Here, we focused on 3 molecules, TP53 has the largest degree as a TF in IA-CFMSN, VEGFA has the largest degree as an immune gene and ESR1 is the only molecule in IA-CFMSN as a TF and an immune gene simultaneously, indicating that these three molecules might play important roles in the immune mechanism of IS.

To further excavate the relationship among the hub genes (PIK3CA, IL6, TNF, and KNG1) of IS immune genes, the main functional pathway (Cytokine-cytokine receptor interaction pathway) and the regulatory motif types (3-node composite FFLs), interestingly, we found that only IL6 was involved in one of the 3-node composite FFLs in IA-CFMSN. Then we retrieved the regulatory relationship of IL6 in Cytokine-cytokine receptor interaction pathway. Therefore, we constructed a regulation map combining a 3-node composite FFL and part of Cytokine-cytokine receptor interaction pathway with IL6 as hub gene to illustrate the relationship among the three (Supplementary Figure S3). Such result suggested that although IL6 does not appear as the most central node in the motifs we identified, we still cannot ignore the important role of IL6 in IS immunomodulation.

### Excavation of Potential Drug Through IA-CFMSN

The 9 immune genes (CXCR4, IL6, CXCL12, FOS, VEGFA, AGTR1, EDN1, FGF2, and AKT1) in IA-CFMSN were used to identify candidate drugs through the DrugBank database. It was found that 20 approved drugs target 5 immune genes (CXCR4, IL6, VEGFA, AGTR1, and FGF2). Eleven of 20 approved drugs target AGTR1, and 10 out of the 11 drugs were Angiotensin II Receptor Blockers (ARBs), one of the most commonly used first-line treatments for hypertension, indicating its potential value in the treatment of IS. The information of drug-gene pairs was shown in Supplementary Table S5. Besides, TFs and miRNAs were also applied to excavate candidate drugs, however, due to some reason, they are not suitable for further analysis, and were discussed in the discussion section.

**FIGURE 6:**
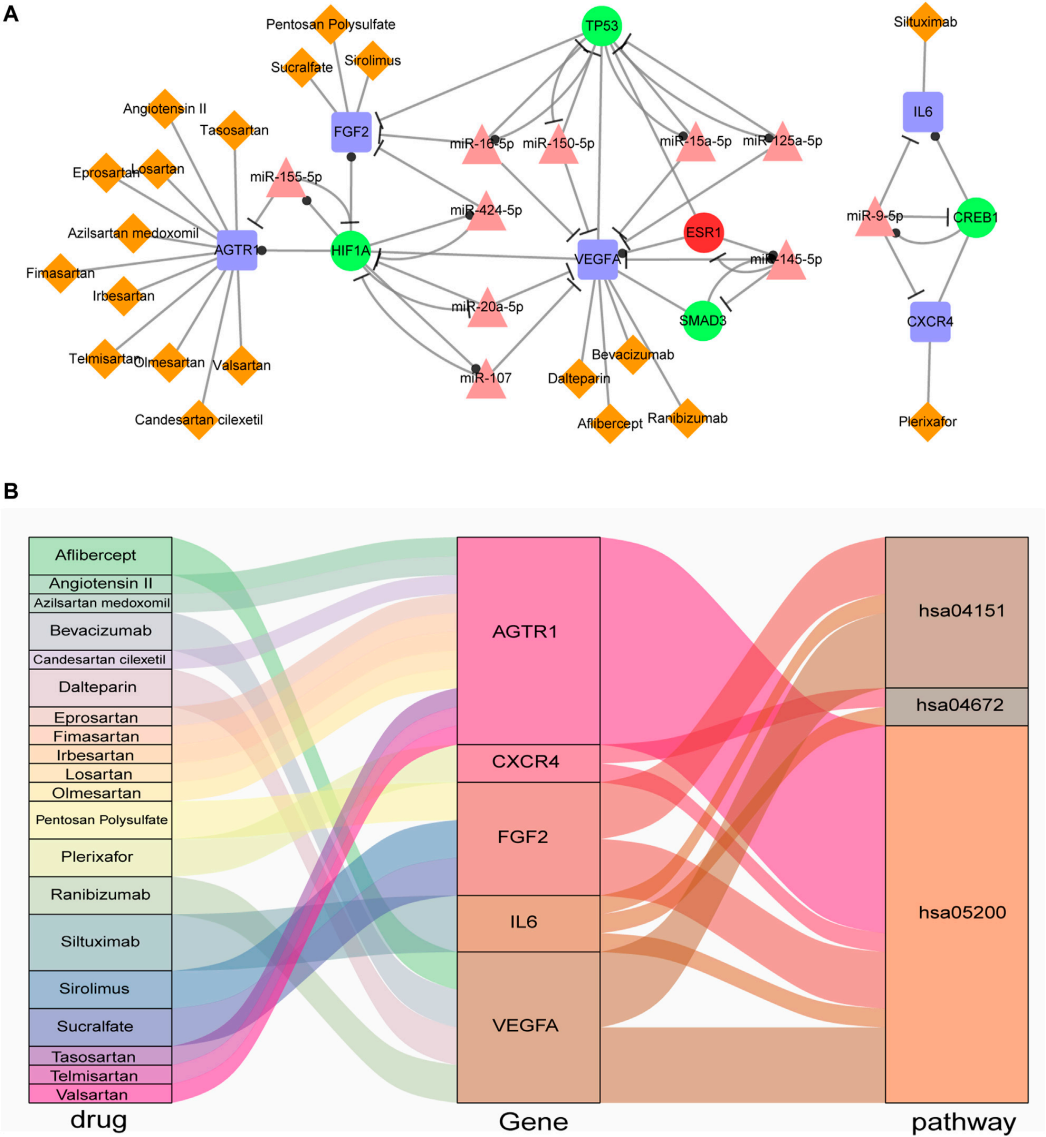
Drugs screened from IA-CFMSN. **(A)** A network of selected drugs targeted with screened immune genes and their FFLs. **(B)** A Sankey diagram of drug-gene-pathway relationships screened from IA-CFMSN.

Above all, we constructed a network containing the 5 immune genes (CXCR4, IL6, VEGFA, AGTR1, and FGF2) and their relevance FFLs with drug-gene target pairs and was visualized in Figure 6A. In addition, to further figure out the characteristic of these 5 immune genes in IA-CFMSN, we made a KEGG enrichment analysis, as a result, 3 significant pathways were identified: Pathways in cancer (hsa05200), PI3K-Akt signaling pathway (hsa04151), and Intestinal immune network for IgA production (hsa04672). Then a Sankey diagram of the drug-gene-pathway was constructed to better observe their relationships (Figure 6B). For example, Ranibizumab can target VEGFA and regulate the two pathways of hsa04151 and hsa05200 to play the role of immunomodulation after ischemic stroke, and may provide a basis for the following mechanism research.

## Discussion

At present, although recanalization treatment of cerebral infarction has achieved a good therapeutic effect, however, researchers are still exploring whether there are new neuroprotective methods to reduce inflammatory response after stroke and its mechanisms. In our present study, immune genes of ischemic stroke were discovered and the immune-associated composite FFL motif-specific sub-network (IA-CFMSN) of ischemic stroke was constructed to explain the underlying mechanism and excavate potential drugs. Our study will have the potential to provide new insights into the underlying molecular mechanisms and expand the scope of neuroprotective drug candidates for IS.

We first constructed a PPI network using IS immune genes, and PIK3CA (PI3K) was found to be the hub gene of this network, indicating that PIK3CA (PI3K) might play an important role in the immune regulation after ischemic stroke. PI3K is part of the PI3K-AKT signaling pathway, which participates in an emerging mechanism of ischemic stroke. For example, suppression of CXCL8 promotes the activation of microglia and inhibits neuroinflammation by regulating the PI3K/Akt/NF-κB-signaling pathway in IS (Lv et al., 2019). Meanwhile, plenty of new drugs, such as luteolin (Luo et al., 2019), ginsenoside Rg1 (Chen et al., 2019a), and baicalein (Yang et al., 2019) were also found to play a neuroprotective role by targeting the PI3K-Akt signaling pathway after ischemic stroke. Erskine Chu et al. summarized that dysregulated PI3K-AKT signaling in microglia could shape chronic neuroinflammation, and put forward the view that pharmacological manipulation of the PI3K-AKT signaling axis can yield neuroprotective effects based on various studies (Chu et al., 2021). In addition, other top immune genes are IL6 and TNF, which are also important immunoinflammatory molecules that participated in ischemic stroke (Chen et al., 2019b; Jenny et al., 2019). For example, a REGARDS cohort study suggested that IL6 was the only inflammatory cytokine to be strongly associated with the risk of stroke (Jenny et al., 2019); a meta-analysis revealed that higher circulating IL6 levels are associated with higher long-term risk of incident IS independent of conventional vascular risk factors (Papadopoulos et al., 2021). TNF was reported to be produced by microglia and leukocytes in ischemic stroke tissue and plasma TNF receptor 1 (TNFR1) and TNFR2 target post-stroke inflammation could be a promising adjunctive therapy for IS patients (Clausen et al., 2020); however, Kang X et al. held a different view that no protective effect of TNF/TNFR1 inhibition on IS was observed through drug-target Mendelian randomization (Kang et al., 2022). Although the function of PI3K, IL6 and TNF in neuroinflammation after IS needs further investigation, the above information is sufficient to convince us that they play a key role in neuroinflammation after IS.

Furthermore, pathway enrichment analysis performed using immune genes of cluster 1 and cluster 2 generated from the PPI network demonstrated the role of immune genes in elucidating the mechanism of IS. Based on the results, the potentially significant role of the cytokine-cytokine receptor interaction pathway in IS was further explored. Chemokines (CC and CXC subfamily), interleukins, TGF-β family, and TNF family presented a prominent position in this pathway, which is in accordance with the above results that IL6 and TNF were hub genes and might function as star molecules in regulating immune response following ischemic stroke. However, IL6 is neither in cluster 1 (Figure 1B) nor in cluster 2 (Figure 1C), suggesting that IL6 may not function in complexes with other molecules.

The reasons why we chose FANMOD to detect network motifs were listed in our previous work (Bo et al., 2021). Therefore, in this research, we continue to use FANMOD to detect 3-node motifs, which is suitable and credible. The construction of IA-CFMSN provided an intuitive view of the relationship of TFs, miRNAs, and immune genes for IS. IA-CFMSN contained 21 3-node FFLs composed of 13 miRNAs, 4 TFs, 9 immune genes, and 1 TF& immune gene, among which TP53 regulated 9 out of 21 identified FFLs, and VEGFA participated 8 out of 21 identified FFLs, respectively. Meanwhile, four FFLs are simultaneously composed of both TP53 and VEGFA, which suggested that as a transcription factor, TP53 might regulate the immune response in the central nervous system through regulating VEGFA. The p53 protein is an important transcriptional regulator of apoptosis, and Tp53 Arg72Pro polymorphism was reported to govern neuronal vulnerability to apoptosis after stroke (Gomez-Sanchez et al., 2011). Neutralization of IL-9 ameliorates post-stroke damage to the blood-brain barrier (BBB) via down-regulating astrocyte-derived VEGF-A, which suggested VEGFA as a novel therapeutic target of immune intervention of IS (Tan et al., 2019). It has been reported that TP53 has a close connection with VEGFA in cancer researches, for example, TP53 mutations are a marker of increased VEGF expression, and can predict sensitivity to VEGF/VEGFR inhibitors in cancer treatment (Wheler et al., 2016; Li et al., 2020). However, the exact mechanism of the effect of TP53 on VEGFA in ischemic stroke remains to be further explored. Nampoothiri SS et al. (Nampoothiri et al., 2018) identified two key TFs, namely NFkB and STAT, with a similar approach using 5 node FFLs. Nampoothiri SS et al. mentioned that STAT was reported to participate in neuronal survival and regeneration (Dziennis and Alkayed, 2008), and NF-kB induces proinflammatory factors, producing innate inflammation and regulated apoptosis (Harari and Liao, 2010), suggesting its role in neuroinflammation. However, these 2 TFs were not discovered in our present analysis, mainly due to a different screening method for genes associated with IS. NF-kB and STAT are genes related to IS with relevance scores less than 10, therefore, they did not make the list of IS-related genes in our study.

Through IA-CFMSN, we hope to investigate candidate drugs targeting TFs, miRNAs, and immune genes in this network. However, TFs and miRNAs were found to be not suitable for digging out drugs as we expected. The 4 TFs (CREB1, HIF1A, SMAD3, and TP53) and 1 TF& immune gene (ESR1) in IA-CFMSN were applied to identify candidate drugs. As a result, only 38 drugs targeting ESR1 were identified (Supplementary Table S5). However, the drugs that target ESR1 are mainly estrogen, progesterone, selective estrogen receptor modulators, etc., and the main cause of ischemic stroke is due to vascular obstruction. Although this type of estrogen and progesterone drugs can play a role in immune regulation and may have the effect of inhibiting the immune response after infarction to a certain extent, this type of drug is not suitable for applications such as cerebral infarction where the proportion of men and women is roughly equal. Therefore, the drugs target ESR1 are not suitable for the treatment of IS patients, so they are excluded from the current study. Furthermore, we also intended to figure out whether miRNA identified from IA-CFMSN can affect drugs in IS. Thus, miRNA-drug pairs were obtained by merging miRNA-gene pairs and drug-gene pairs. Then, the drugs with less than 2 target genes were discarded. Finally, significant miRNA-drug pairs were discovered using the cumulative hypergeometric distribution. We believed that it was statistically significant if *p*-value was less than 0.05. However, we have not screened out the miRNA-drug relationship pairs that meet the above conditions (Supplementary Table S6).

Therefore, we screened 5 immune genes targeted by 20 drugs, among which, 11 drugs target AGTR1. Ten out of the 11 drugs were Angiotensin II Receptor Blockers (ARBs), one of the most commonly used first-line treatments for hypertension. Drugs (ARBs or sartans) that block angiotensin II type 1 receptors (AT1R, encoded by AGTR1) are strongly neuroprotective, neurorestorative and anti-inflammatory (Villapol et al., 2015). It was reported that telmisartan can greatly reduce persistent inflammation and protect the neurovascular unit after ischemic stroke in hypertensive rats (Kono et al., 2015). Besides, Candesartan can affect brain endothelial cells, upregulated VEGF-A and VEGF-B, and exert a paracrine neuroprotective effect (Soliman et al., 2014; Ishrat et al., 2018). Losartan can protect against cerebral ischemia/reperfusion-induced apoptosis through β-arrestin1-mediated phosphorylation of Akt (Chen et al., 2017). Allisartan isoproxil, a novel ARB developed in China, can be used for stroke prevention via BP reduction and organ protection, with the molecular mechanisms related to inhibition of angiotensin-aldosterone system and oxidative stress (Ling et al., 2021). For clinical data, Pai’s et al. found that in the “real world” practice in patients with hypertension and diabetes, those treated with ARBs regimen reduces 26% of stroke in contrast to the group with angiotensin converting enzyme inhibitors (ACEIs) regimen (Pai et al., 2016). These indicate that in addition to controlling the risk factors of cerebrovascular disease by reducing blood pressure, ARB can also play a neuroprotective role by controlling inflammation, endothelial cells, and apoptosis, which will also help us pay more attention to ARB in the treatment of ischemic stroke in the future.

## Conclusion

In conclusion, we systematically identified potential hub immune genes (PI3K, IL6, and TNF) and unfolded the Cytokine-cytokine receptor interaction pathway as the basic immune mechanism of IS. Then, we constructed IA-CFMSN, screened 21 significant 3-node FFLs in IS, and filtered ARBs through IA-CFMSN as potential drugs to provide new insight into the neuroprotective mechanisms for IS.

## Data Availability

The original contributions presented in the study are included in the article/Supplementary Material, further inquiries can be directed to the corresponding authors.

## Supplementary Material

Supplementary Figure S1 Distribution of immune genes for ischemic stroke.

Supplementary Figure S2 The basic topological features of IA-CFMSN.

Supplementary Figure S3 The regulatory relationship of a 3-node composite FFL and part of Cytokine-cytokine receptor interaction pathway with IL6 as hub gene.
