# Supplementary material for: Construction Immune Related Feed-Forward Loop Network Reveals Angiotensin II Receptor Blocker as Potential Neuroprotective Drug for Ischemic Stroke
Source: Front Genet. 2022 Mar 28;13:811571. doi: 10.3389/fgene.2022.811571 (PMC8995882; doi:10.3389/fgene.2022.811571)
Supplement: Supplementary file 1 [file Table1.DOCX]

**Table S1 Ischemic stroke related genes with a relevance score＞10.**

| Gene Symbol | Description | Category | GC Id | Relevance score | GeneCards Link |
| --- | --- | --- | --- | --- | --- |
| F5 | Coagulation Factor V | Protein Coding | GC01M169511 | 99.68011 | https://www.genecards.org/cgi-bin/carddisp.pl?gene=F5 |
| F2 | Coagulation Factor II, Thrombin | Protein Coding | GC11P046720 | 95.59432 | https://www.genecards.org/cgi-bin/carddisp.pl?gene=F2 |
| NOTCH3 | Notch Receptor 3 | Protein Coding | GC19M015159 | 88.1479 | https://www.genecards.org/cgi-bin/carddisp.pl?gene=NOTCH3 |
| NOS3 | Nitric Oxide Synthase 3 | Protein Coding | GC07P150990 | 86.89293 | https://www.genecards.org/cgi-bin/carddisp.pl?gene=NOS3 |
| MTHFR | Methylenetetrahydrofolate Reductase | Protein Coding | GC01M011785 | 79.48745 | https://www.genecards.org/cgi-bin/carddisp.pl?gene=MTHFR |
| ACE | Angiotensin I Converting Enzyme | Protein Coding | GC17P063477 | 75.96592 | https://www.genecards.org/cgi-bin/carddisp.pl?gene=ACE |
| ALOX5AP | Arachidonate 5-Lipoxygenase Activating Protein | Protein Coding | GC13P030713 | 66.69591 | https://www.genecards.org/cgi-bin/carddisp.pl?gene=ALOX5AP |
| APOE | Apolipoprotein E | Protein Coding | GC19P044906 | 58.42609 | https://www.genecards.org/cgi-bin/carddisp.pl?gene=APOE |
| GP1BA | Glycoprotein Ib Platelet Subunit Alpha | Protein Coding | GC17P004932 | 58.29651 | https://www.genecards.org/cgi-bin/carddisp.pl?gene=GP1BA |
| CRP | C-Reactive Protein | Protein Coding | GC01M159717 | 57.3718 | https://www.genecards.org/cgi-bin/carddisp.pl?gene=CRP |
| LDLR | Low Density Lipoprotein Receptor | Protein Coding | GC19P011091 | 55.33772 | https://www.genecards.org/cgi-bin/carddisp.pl?gene=LDLR |
| PRKCH | Protein Kinase C Eta | Protein Coding | GC14P061187 | 55.23518 | https://www.genecards.org/cgi-bin/carddisp.pl?gene=PRKCH |
| FBN1 | Fibrillin 1 | Protein Coding | GC15M048408 | 53.60833 | https://www.genecards.org/cgi-bin/carddisp.pl?gene=FBN1 |
| IL6 | Interleukin 6 | Protein Coding | GC07P022725 | 50.2401 | https://www.genecards.org/cgi-bin/carddisp.pl?gene=IL6 |
| TNF | Tumor Necrosis Factor | Protein Coding | GC06P049352 | 48.23476 | https://www.genecards.org/cgi-bin/carddisp.pl?gene=TNF |
| SERPINE1 | Serpin Family E Member 1 | Protein Coding | GC07P101127 | 47.20306 | https://www.genecards.org/cgi-bin/carddisp.pl?gene=SERPINE1 |
| PDE4D | Phosphodiesterase 4D | Protein Coding | GC05M058969 | 44.64021 | https://www.genecards.org/cgi-bin/carddisp.pl?gene=PDE4D |
| PIK3CA | Phosphatidylinositol-4,5-Bisphosphate 3-Kinase Catalytic Subunit Alpha | Protein Coding | GC03P179148 | 44.62831 | https://www.genecards.org/cgi-bin/carddisp.pl?gene=PIK3CA |
| PLAT | Plasminogen Activator, Tissue Type | Protein Coding | GC08M042174 | 43.66195 | https://www.genecards.org/cgi-bin/carddisp.pl?gene=PLAT |
| AGTR1 | Angiotensin II Receptor Type 1 | Protein Coding | GC03P148697 | 43.3597 | https://www.genecards.org/cgi-bin/carddisp.pl?gene=AGTR1 |
| NPPB | Natriuretic Peptide B | Protein Coding | GC01M011858 | 43.0883 | https://www.genecards.org/cgi-bin/carddisp.pl?gene=NPPB |
| MMP9 | Matrix Metallopeptidase 9 | Protein Coding | GC20P046008 | 41.67414 | https://www.genecards.org/cgi-bin/carddisp.pl?gene=MMP9 |
| COL4A1 | Collagen Type IV Alpha 1 Chain | Protein Coding | GC13M110148 | 41.33137 | https://www.genecards.org/cgi-bin/carddisp.pl?gene=COL4A1 |
| SELP | Selectin P | Protein Coding | GC01M169558 | 40.13608 | https://www.genecards.org/cgi-bin/carddisp.pl?gene=SELP |
| PON1 | Paraoxonase 1 | Protein Coding | GC07M095297 | 38.98135 | https://www.genecards.org/cgi-bin/carddisp.pl?gene=PON1 |
| AGT | Angiotensinogen | Protein Coding | GC01M230702 | 37.66718 | https://www.genecards.org/cgi-bin/carddisp.pl?gene=AGT |
| NPPA | Natriuretic Peptide A | Protein Coding | GC01M011846 | 37.60857 | https://www.genecards.org/cgi-bin/carddisp.pl?gene=NPPA |
| ACSL4 | Acyl-CoA Synthetase Long Chain Family Member 4 | Protein Coding | GC0XM109624 | 37.23539 | https://www.genecards.org/cgi-bin/carddisp.pl?gene=ACSL4 |
| IL1B | Interleukin 1 Beta | Protein Coding | GC02M112829 | 36.46056 | https://www.genecards.org/cgi-bin/carddisp.pl?gene=IL1B |
| VWF | Von Willebrand Factor | Protein Coding | GC12M005917 | 36.25253 | https://www.genecards.org/cgi-bin/carddisp.pl?gene=VWF |
| ENG | Endoglin | Protein Coding | GC09M127815 | 36.00565 | https://www.genecards.org/cgi-bin/carddisp.pl?gene=ENG |
| MT-ND1 | Mitochondrially Encoded NADH:Ubiquinone Oxidoreductase Core Subunit 1 | Protein Coding | GCMTP003309 | 34.83567 | https://www.genecards.org/cgi-bin/carddisp.pl?gene=MT-ND1 |
| VEGFA | Vascular Endothelial Growth Factor A | Protein Coding | GC06P043770 | 34.4998 | https://www.genecards.org/cgi-bin/carddisp.pl?gene=VEGFA |
| ITGB3 | Integrin Subunit Beta 3 | Protein Coding | GC17P047254 | 34.02005 | https://www.genecards.org/cgi-bin/carddisp.pl?gene=ITGB3 |
| BDNF | Brain Derived Neurotrophic Factor | Protein Coding | GC11M027654 | 33.65009 | https://www.genecards.org/cgi-bin/carddisp.pl?gene=BDNF |
| SERPINC1 | Serpin Family C Member 1 | Protein Coding | GC01M174189 | 33.63627 | https://www.genecards.org/cgi-bin/carddisp.pl?gene=SERPINC1 |
| APOH | Apolipoprotein H | Protein Coding | GC17M066212 | 33.52266 | https://www.genecards.org/cgi-bin/carddisp.pl?gene=APOH |
| APOB | Apolipoprotein B | Protein Coding | GC02M020956 | 31.19627 | https://www.genecards.org/cgi-bin/carddisp.pl?gene=APOB |
| FGB | Fibrinogen Beta Chain | Protein Coding | GC04P154566 | 30.97505 | https://www.genecards.org/cgi-bin/carddisp.pl?gene=FGB |
| FGA | Fibrinogen Alpha Chain | Protein Coding | GC04M154583 | 30.73417 | https://www.genecards.org/cgi-bin/carddisp.pl?gene=FGA |
| LPA | Lipoprotein(A) | Protein Coding | GC06M160531 | 30.22858 | https://www.genecards.org/cgi-bin/carddisp.pl?gene=LPA |
| MT-ND5 | Mitochondrially Encoded NADH:Ubiquinone Oxidoreductase Core Subunit 5 | Protein Coding | GCMTP012339 | 30.14733 | https://www.genecards.org/cgi-bin/carddisp.pl?gene=MT-ND5 |
| F7 | Coagulation Factor VII | Protein Coding | GC13P113105 | 29.72815 | https://www.genecards.org/cgi-bin/carddisp.pl?gene=F7 |
| IL10 | Interleukin 10 | Protein Coding | GC01M206767 | 29.55642 | https://www.genecards.org/cgi-bin/carddisp.pl?gene=IL10 |
| CST3 | Cystatin C | Protein Coding | GC20M023627 | 29.53977 | https://www.genecards.org/cgi-bin/carddisp.pl?gene=CST3 |
| F3 | Coagulation Factor III, Tissue Factor | Protein Coding | GC01M094530 | 29.09203 | https://www.genecards.org/cgi-bin/carddisp.pl?gene=F3 |
| GLA | Galactosidase Alpha | Protein Coding | GC0XM101393 | 28.75051 | https://www.genecards.org/cgi-bin/carddisp.pl?gene=GLA |
| ACTA2 | Actin Alpha 2, Smooth Muscle | Protein Coding | GC10M088935 | 28.29706 | https://www.genecards.org/cgi-bin/carddisp.pl?gene=ACTA2 |
| EDN1 | Endothelin 1 | Protein Coding | GC06P012256 | 28.1782 | https://www.genecards.org/cgi-bin/carddisp.pl?gene=EDN1 |
| TLR4 | Toll Like Receptor 4 | Protein Coding | GC09P117704 | 27.58714 | https://www.genecards.org/cgi-bin/carddisp.pl?gene=TLR4 |
| ADAMTS13 | ADAM Metallopeptidase With Thrombospondin Type 1 Motif 13 | Protein Coding | GC09P133414 | 27.57772 | https://www.genecards.org/cgi-bin/carddisp.pl?gene=ADAMTS13 |
| CXCL12 | C-X-C Motif Chemokine Ligand 12 | Protein Coding | GC10M044294 | 27.38297 | https://www.genecards.org/cgi-bin/carddisp.pl?gene=CXCL12 |
| MT-ND4 | Mitochondrially Encoded NADH:Ubiquinone Oxidoreductase Core Subunit 4 | Protein Coding | GCMTP010762 | 27.29738 | https://www.genecards.org/cgi-bin/carddisp.pl?gene=MT-ND4 |
| MT-ND6 | Mitochondrially Encoded NADH:Ubiquinone Oxidoreductase Core Subunit 6 | Protein Coding | GCMTM014151 | 27.28711 | https://www.genecards.org/cgi-bin/carddisp.pl?gene=MT-ND6 |
| PLA2G7 | Phospholipase A2 Group VII | Protein Coding | GC06M046704 | 27.28623 | https://www.genecards.org/cgi-bin/carddisp.pl?gene=PLA2G7 |
| SELE | Selectin E | Protein Coding | GC01M169722 | 26.96853 | https://www.genecards.org/cgi-bin/carddisp.pl?gene=SELE |
| ICAM1 | Intercellular Adhesion Molecule 1 | Protein Coding | GC19P010270 | 26.84697 | https://www.genecards.org/cgi-bin/carddisp.pl?gene=ICAM1 |
| JAK2 | Janus Kinase 2 | Protein Coding | GC09P004985 | 26.53889 | https://www.genecards.org/cgi-bin/carddisp.pl?gene=JAK2 |
| PON2 | Paraoxonase 2 | Protein Coding | GC07M095404 | 26.44621 | https://www.genecards.org/cgi-bin/carddisp.pl?gene=PON2 |
| ADA2 | Adenosine Deaminase 2 | Protein Coding | GC22M017179 | 26.16538 | https://www.genecards.org/cgi-bin/carddisp.pl?gene=ADA2 |
| THBD | Thrombomodulin | Protein Coding | GC20M023026 | 25.96107 | https://www.genecards.org/cgi-bin/carddisp.pl?gene=THBD |
| HTRA1 | HtrA Serine Peptidase 1 | Protein Coding | GC10P122461 | 25.81661 | https://www.genecards.org/cgi-bin/carddisp.pl?gene=HTRA1 |
| ADIPOQ | Adiponectin, C1Q And Collagen Domain Containing | Protein Coding | GC03P186842 | 25.59866 | https://www.genecards.org/cgi-bin/carddisp.pl?gene=ADIPOQ |
| APP | Amyloid Beta Precursor Protein | Protein Coding | GC21M025880 | 25.57547 | https://www.genecards.org/cgi-bin/carddisp.pl?gene=APP |
| MAPT | Microtubule Associated Protein Tau | Protein Coding | GC17P045894 | 25.38535 | https://www.genecards.org/cgi-bin/carddisp.pl?gene=MAPT |
| CASP3 | Caspase 3 | Protein Coding | GC04M184627 | 25.37456 | https://www.genecards.org/cgi-bin/carddisp.pl?gene=CASP3 |
| LPL | Lipoprotein Lipase | Protein Coding | GC08P019901 | 25.10884 | https://www.genecards.org/cgi-bin/carddisp.pl?gene=LPL |
| RNF213 | Ring Finger Protein 213 | Protein Coding | GC17P080260 | 25.03992 | https://www.genecards.org/cgi-bin/carddisp.pl?gene=RNF213 |
| REN | Renin | Protein Coding | GC01M204154 | 25.0218 | https://www.genecards.org/cgi-bin/carddisp.pl?gene=REN |
| ALB | Albumin | Protein Coding | GC04P073397 | 24.88259 | https://www.genecards.org/cgi-bin/carddisp.pl?gene=ALB |
| TP53 | Tumor Protein P53 | Protein Coding | GC17M007661 | 24.82591 | https://www.genecards.org/cgi-bin/carddisp.pl?gene=TP53 |
| ENO2 | Enolase 2 | Protein Coding | GC12P006913 | 24.81312 | https://www.genecards.org/cgi-bin/carddisp.pl?gene=ENO2 |
| MT-ATP6 | Mitochondrially Encoded ATP Synthase Membrane Subunit 6 | Protein Coding | GCMTP008531 | 24.59047 | https://www.genecards.org/cgi-bin/carddisp.pl?gene=MT-ATP6 |
| PTGS2 | Prostaglandin-Endoperoxide Synthase 2 | Protein Coding | GC01M186640 | 24.41143 | https://www.genecards.org/cgi-bin/carddisp.pl?gene=PTGS2 |
| HIF1A | Hypoxia Inducible Factor 1 Subunit Alpha | Protein Coding | GC14P061695 | 24.25867 | https://www.genecards.org/cgi-bin/carddisp.pl?gene=HIF1A |
| F13A1 | Coagulation Factor XIII A Chain | Protein Coding | GC06M006144 | 23.8203 | https://www.genecards.org/cgi-bin/carddisp.pl?gene=F13A1 |
| COL4A2 | Collagen Type IV Alpha 2 Chain | Protein Coding | GC13P110305 | 23.79203 | https://www.genecards.org/cgi-bin/carddisp.pl?gene=COL4A2 |
| VKORC1 | Vitamin K Epoxide Reductase Complex Subunit 1 | Protein Coding | GC16M031105 | 23.46769 | https://www.genecards.org/cgi-bin/carddisp.pl?gene=VKORC1 |
| CYP2C19 | Cytochrome P450 Family 2 Subfamily C Member 19 | Protein Coding | GC10P094762 | 23.40204 | https://www.genecards.org/cgi-bin/carddisp.pl?gene=CYP2C19 |
| APOA1 | Apolipoprotein A1 | Protein Coding | GC11M116835 | 23.31786 | https://www.genecards.org/cgi-bin/carddisp.pl?gene=APOA1 |
| CCL2 | C-C Motif Chemokine Ligand 2 | Protein Coding | GC17P034255 | 23.23381 | https://www.genecards.org/cgi-bin/carddisp.pl?gene=CCL2 |
| EDNRA | Endothelin Receptor Type A | Protein Coding | GC04P147480 | 23.18409 | https://www.genecards.org/cgi-bin/carddisp.pl?gene=EDNRA |
| F10 | Coagulation Factor X | Protein Coding | GC13P113122 | 23.17652 | https://www.genecards.org/cgi-bin/carddisp.pl?gene=F10 |
| SOD2 | Superoxide Dismutase 2 | Protein Coding | GC06M159669 | 23.00794 | https://www.genecards.org/cgi-bin/carddisp.pl?gene=SOD2 |
| MT-CO1 | Mitochondrially Encoded Cytochrome C Oxidase I | Protein Coding | GCMTP005906 | 22.67818 | https://www.genecards.org/cgi-bin/carddisp.pl?gene=MT-CO1 |
| PROZ | Protein Z, Vitamin K Dependent Plasma Glycoprotein | Protein Coding | GC13P113158 | 22.54769 | https://www.genecards.org/cgi-bin/carddisp.pl?gene=PROZ |
| IL1RN | Interleukin 1 Receptor Antagonist | Protein Coding | GC02P115722 | 22.47127 | https://www.genecards.org/cgi-bin/carddisp.pl?gene=IL1RN |
| HMGCR | 3-Hydroxy-3-Methylglutaryl-CoA Reductase | Protein Coding | GC05P075336 | 22.32961 | https://www.genecards.org/cgi-bin/carddisp.pl?gene=HMGCR |
| PPARG | Peroxisome Proliferator Activated Receptor Gamma | Protein Coding | GC03P012287 | 22.25574 | https://www.genecards.org/cgi-bin/carddisp.pl?gene=PPARG |
| ITGA2 | Integrin Subunit Alpha 2 | Protein Coding | GC05P052989 | 22.25359 | https://www.genecards.org/cgi-bin/carddisp.pl?gene=ITGA2 |
| SERPINA3 | Serpin Family A Member 3 | Protein Coding | GC14P094612 | 22.01918 | https://www.genecards.org/cgi-bin/carddisp.pl?gene=SERPINA3 |
| OLR1 | Oxidized Low Density Lipoprotein Receptor 1 | Protein Coding | GC12M014384 | 21.99376 | https://www.genecards.org/cgi-bin/carddisp.pl?gene=OLR1 |
| ADRB2 | Adrenoceptor Beta 2 | Protein Coding | GC05P148825 | 21.96506 | https://www.genecards.org/cgi-bin/carddisp.pl?gene=ADRB2 |
| VCAM1 | Vascular Cell Adhesion Molecule 1 | Protein Coding | GC01P100719 | 21.87615 | https://www.genecards.org/cgi-bin/carddisp.pl?gene=VCAM1 |
| ABCA1 | ATP Binding Cassette Subfamily A Member 1 | Protein Coding | GC09M104781 | 21.83108 | https://www.genecards.org/cgi-bin/carddisp.pl?gene=ABCA1 |
| SOD1 | Superoxide Dismutase 1 | Protein Coding | GC21P031659 | 21.83017 | https://www.genecards.org/cgi-bin/carddisp.pl?gene=SOD1 |
| GUCY1A1 | Guanylate Cyclase 1 Soluble Subunit Alpha 1 | Protein Coding | GC04P155667 | 21.80466 | https://www.genecards.org/cgi-bin/carddisp.pl?gene=GUCY1A1 |
| ADM | Adrenomedullin | Protein Coding | GC11P010304 | 21.71121 | https://www.genecards.org/cgi-bin/carddisp.pl?gene=ADM |
| P2RY12 | Purinergic Receptor P2Y12 | Protein Coding | GC03M151336 | 21.62217 | https://www.genecards.org/cgi-bin/carddisp.pl?gene=P2RY12 |
| F2R | Coagulation Factor II Thrombin Receptor | Protein Coding | GC05P076716 | 21.54122 | https://www.genecards.org/cgi-bin/carddisp.pl?gene=F2R |
| PTGIS | Prostaglandin I2 Synthase | Protein Coding | GC20M049503 | 21.52918 | https://www.genecards.org/cgi-bin/carddisp.pl?gene=PTGIS |
| INS | Insulin | Protein Coding | GC11M002159 | 21.48502 | https://www.genecards.org/cgi-bin/carddisp.pl?gene=INS |
| PECAM1 | Platelet And Endothelial Cell Adhesion Molecule 1 | Protein Coding | GC17M064319 | 21.46414 | https://www.genecards.org/cgi-bin/carddisp.pl?gene=PECAM1 |
| MT-CO2 | Mitochondrially Encoded Cytochrome C Oxidase II | Protein Coding | GCMTP007587 | 21.07789 | https://www.genecards.org/cgi-bin/carddisp.pl?gene=MT-CO2 |
| TNNT2 | Troponin T2, Cardiac Type | Protein Coding | GC01M201359 | 20.94298 | https://www.genecards.org/cgi-bin/carddisp.pl?gene=TNNT2 |
| IGF1 | Insulin Like Growth Factor 1 | Protein Coding | GC12M102395 | 20.8679 | https://www.genecards.org/cgi-bin/carddisp.pl?gene=IGF1 |
| ALOX5 | Arachidonate 5-Lipoxygenase | Protein Coding | GC10P045374 | 20.76879 | https://www.genecards.org/cgi-bin/carddisp.pl?gene=ALOX5 |
| MMP3 | Matrix Metallopeptidase 3 | Protein Coding | GC11M102835 | 20.57675 | https://www.genecards.org/cgi-bin/carddisp.pl?gene=MMP3 |
| CXCL8 | C-X-C Motif Chemokine Ligand 8 | Protein Coding | GC04P073740 | 20.42648 | https://www.genecards.org/cgi-bin/carddisp.pl?gene=CXCL8 |
| GNB3 | G Protein Subunit Beta 3 | Protein Coding | GC12P006839 | 20.24396 | https://www.genecards.org/cgi-bin/carddisp.pl?gene=GNB3 |
| PRKG1 | Protein Kinase CGMP-Dependent 1 | Protein Coding | GC10P050991 | 20.1213 | https://www.genecards.org/cgi-bin/carddisp.pl?gene=PRKG1 |
| KNG1 | Kininogen 1 | Protein Coding | GC03P186717 | 20.04958 | https://www.genecards.org/cgi-bin/carddisp.pl?gene=KNG1 |
| ITGA2B | Integrin Subunit Alpha 2b | Protein Coding | GC17M044399 | 20.03411 | https://www.genecards.org/cgi-bin/carddisp.pl?gene=ITGA2B |
| ESR1 | Estrogen Receptor 1 | Protein Coding | GC06P151656 | 19.95045 | https://www.genecards.org/cgi-bin/carddisp.pl?gene=ESR1 |
| ANGPT1 | Angiopoietin 1 | Protein Coding | GC08M107246 | 19.93453 | https://www.genecards.org/cgi-bin/carddisp.pl?gene=ANGPT1 |
| ADD1 | Adducin 1 | Protein Coding | GC04P002855 | 19.77495 | https://www.genecards.org/cgi-bin/carddisp.pl?gene=ADD1 |
| HMGB1 | High Mobility Group Box 1 | Protein Coding | GC13M030456 | 19.76057 | https://www.genecards.org/cgi-bin/carddisp.pl?gene=HMGB1 |
| GSR | Glutathione-Disulfide Reductase | Protein Coding | GC08M030678 | 19.57478 | https://www.genecards.org/cgi-bin/carddisp.pl?gene=GSR |
| CD40LG | CD40 Ligand | Protein Coding | GC0XP136649 | 19.52918 | https://www.genecards.org/cgi-bin/carddisp.pl?gene=CD40LG |
| ELN | Elastin | Protein Coding | GC07P074027 | 19.51324 | https://www.genecards.org/cgi-bin/carddisp.pl?gene=ELN |
| MMP2 | Matrix Metallopeptidase 2 | Protein Coding | GC16P055390 | 19.50015 | https://www.genecards.org/cgi-bin/carddisp.pl?gene=MMP2 |
| NGB | Neuroglobin | Protein Coding | GC14M077265 | 19.30902 | https://www.genecards.org/cgi-bin/carddisp.pl?gene=NGB |
| PF4 | Platelet Factor 4 | Protein Coding | GC04M073980 | 19.27815 | https://www.genecards.org/cgi-bin/carddisp.pl?gene=PF4 |
| ADORA1 | Adenosine A1 Receptor | Protein Coding | GC01P203090 | 19.14089 | https://www.genecards.org/cgi-bin/carddisp.pl?gene=ADORA1 |
| AVP | Arginine Vasopressin | Protein Coding | GC20M003082 | 19.11182 | https://www.genecards.org/cgi-bin/carddisp.pl?gene=AVP |
| TNNI3 | Troponin I3, Cardiac Type | Protein Coding | GC19M055151 | 19.09365 | https://www.genecards.org/cgi-bin/carddisp.pl?gene=TNNI3 |
| NOS2 | Nitric Oxide Synthase 2 | Protein Coding | GC17M027756 | 18.9218 | https://www.genecards.org/cgi-bin/carddisp.pl?gene=NOS2 |
| MT-CO3 | Mitochondrially Encoded Cytochrome C Oxidase III | Protein Coding | GCMTP009209 | 18.90386 | https://www.genecards.org/cgi-bin/carddisp.pl?gene=MT-CO3 |
| CD14 | CD14 Molecule | Protein Coding | GC05M140631 | 18.89735 | https://www.genecards.org/cgi-bin/carddisp.pl?gene=CD14 |
| HSPA4 | Heat Shock Protein Family A (Hsp70) Member 4 | Protein Coding | GC05P133051 | 18.78115 | https://www.genecards.org/cgi-bin/carddisp.pl?gene=HSPA4 |
| S100B | S100 Calcium Binding Protein B | Protein Coding | GC21M047706 | 18.71531 | https://www.genecards.org/cgi-bin/carddisp.pl?gene=S100B |
| PROCR | Protein C Receptor | Protein Coding | GC20P035194 | 18.70024 | https://www.genecards.org/cgi-bin/carddisp.pl?gene=PROCR |
| CREB1 | CAMP Responsive Element Binding Protein 1 | Protein Coding | GC02P207529 | 18.69794 | https://www.genecards.org/cgi-bin/carddisp.pl?gene=CREB1 |
| TGFB1 | Transforming Growth Factor Beta 1 | Protein Coding | GC19M041301 | 18.68356 | https://www.genecards.org/cgi-bin/carddisp.pl?gene=TGFB1 |
| MB | Myoglobin | Protein Coding | GC22M035606 | 18.61547 | https://www.genecards.org/cgi-bin/carddisp.pl?gene=MB |
| LTA | Lymphotoxin Alpha | Protein Coding | GC06P049350 | 18.48619 | https://www.genecards.org/cgi-bin/carddisp.pl?gene=LTA |
| SLC1A2 | Solute Carrier Family 1 Member 2 | Protein Coding | GC11M035252 | 18.39707 | https://www.genecards.org/cgi-bin/carddisp.pl?gene=SLC1A2 |
| CETP | Cholesteryl Ester Transfer Protein | Protein Coding | GC16P056961 | 18.39288 | https://www.genecards.org/cgi-bin/carddisp.pl?gene=CETP |
| AQP4 | Aquaporin 4 | Protein Coding | GC18M026852 | 18.25825 | https://www.genecards.org/cgi-bin/carddisp.pl?gene=AQP4 |
| LOX | Lysyl Oxidase | Protein Coding | GC05M122063 | 18.23831 | https://www.genecards.org/cgi-bin/carddisp.pl?gene=LOX |
| GFAP | Glial Fibrillary Acidic Protein | Protein Coding | GC17M044905 | 18.2037 | https://www.genecards.org/cgi-bin/carddisp.pl?gene=GFAP |
| PIK3C2A | Phosphatidylinositol-4-Phosphate 3-Kinase Catalytic Subunit Type 2 Alpha | Protein Coding | GC11M017211 | 18.14107 | https://www.genecards.org/cgi-bin/carddisp.pl?gene=PIK3C2A |
| EPO | Erythropoietin | Protein Coding | GC07P100720 | 18.118 | https://www.genecards.org/cgi-bin/carddisp.pl?gene=EPO |
| PTGS1 | Prostaglandin-Endoperoxide Synthase 1 | Protein Coding | GC09P122370 | 18.09253 | https://www.genecards.org/cgi-bin/carddisp.pl?gene=PTGS1 |
| MAP2 | Microtubule Associated Protein 2 | Protein Coding | GC02P209424 | 17.9714 | https://www.genecards.org/cgi-bin/carddisp.pl?gene=MAP2 |
| SPP1 | Secreted Phosphoprotein 1 | Protein Coding | GC04P087975 | 17.871 | https://www.genecards.org/cgi-bin/carddisp.pl?gene=SPP1 |
| LMNA | Lamin A/C | Protein Coding | GC01P156082 | 17.75154 | https://www.genecards.org/cgi-bin/carddisp.pl?gene=LMNA |
| FGF2 | Fibroblast Growth Factor 2 | Protein Coding | GC04P122826 | 17.73041 | https://www.genecards.org/cgi-bin/carddisp.pl?gene=FGF2 |
| CBS | Cystathionine Beta-Synthase | Protein Coding | GC21M043053 | 17.62626 | https://www.genecards.org/cgi-bin/carddisp.pl?gene=CBS |
| TTR | Transthyretin | Protein Coding | GC18P031557 | 17.53582 | https://www.genecards.org/cgi-bin/carddisp.pl?gene=TTR |
| CYCS | Cytochrome C, Somatic | Protein Coding | GC07M025118 | 17.49785 | https://www.genecards.org/cgi-bin/carddisp.pl?gene=CYCS |
| GP6 | Glycoprotein VI Platelet | Protein Coding | GC19M055013 | 17.43877 | https://www.genecards.org/cgi-bin/carddisp.pl?gene=GP6 |
| SERPINI1 | Serpin Family I Member 1 | Protein Coding | GC03P167735 | 17.37677 | https://www.genecards.org/cgi-bin/carddisp.pl?gene=SERPINI1 |
| HSPA8 | Heat Shock Protein Family A (Hsp70) Member 8 | Protein Coding | GC11M123057 | 17.25773 | https://www.genecards.org/cgi-bin/carddisp.pl?gene=HSPA8 |
| EPHX2 | Epoxide Hydrolase 2 | Protein Coding | GC08P027490 | 17.23292 | https://www.genecards.org/cgi-bin/carddisp.pl?gene=EPHX2 |
| AKT1 | AKT Serine/Threonine Kinase 1 | Protein Coding | GC14M104769 | 17.16527 | https://www.genecards.org/cgi-bin/carddisp.pl?gene=AKT1 |
| IL1A | Interleukin 1 Alpha | Protein Coding | GC02M112773 | 17.14256 | https://www.genecards.org/cgi-bin/carddisp.pl?gene=IL1A |
| GJA1 | Gap Junction Protein Alpha 1 | Protein Coding | GC06P121436 | 17.13552 | https://www.genecards.org/cgi-bin/carddisp.pl?gene=GJA1 |
| COL3A1 | Collagen Type III Alpha 1 Chain | Protein Coding | GC02P188974 | 17.12651 | https://www.genecards.org/cgi-bin/carddisp.pl?gene=COL3A1 |
| ADAMTS1 | ADAM Metallopeptidase With Thrombospondin Type 1 Motif 1 | Protein Coding | GC21M026835 | 17.10512 | https://www.genecards.org/cgi-bin/carddisp.pl?gene=ADAMTS1 |
| NES | Nestin | Protein Coding | GC01M156668 | 17.02674 | https://www.genecards.org/cgi-bin/carddisp.pl?gene=NES |
| PPBP | Pro-Platelet Basic Protein | Protein Coding | GC04M073986 | 16.985 | https://www.genecards.org/cgi-bin/carddisp.pl?gene=PPBP |
| IL18 | Interleukin 18 | Protein Coding | GC11M112143 | 16.95729 | https://www.genecards.org/cgi-bin/carddisp.pl?gene=IL18 |
| IL4 | Interleukin 4 | Protein Coding | GC05P132673 | 16.8777 | https://www.genecards.org/cgi-bin/carddisp.pl?gene=IL4 |
| HMOX1 | Heme Oxygenase 1 | Protein Coding | GC22P035380 | 16.74549 | https://www.genecards.org/cgi-bin/carddisp.pl?gene=HMOX1 |
| SULT1A3 | Sulfotransferase Family 1A Member 3 | Protein Coding | GC16P030199 | 16.72922 | https://www.genecards.org/cgi-bin/carddisp.pl?gene=SULT1A3 |
| PLG | Plasminogen | Protein Coding | GC06P160702 | 16.61137 | https://www.genecards.org/cgi-bin/carddisp.pl?gene=PLG |
| CXCR4 | C-X-C Motif Chemokine Receptor 4 | Protein Coding | GC02M136114 | 16.52672 | https://www.genecards.org/cgi-bin/carddisp.pl?gene=CXCR4 |
| EDNRB | Endothelin Receptor Type B | Protein Coding | GC13M077895 | 16.4971 | https://www.genecards.org/cgi-bin/carddisp.pl?gene=EDNRB |
| ENPP1 | Ectonucleotide Pyrophosphatase/Phosphodiesterase 1 | Protein Coding | GC06P131808 | 16.44648 | https://www.genecards.org/cgi-bin/carddisp.pl?gene=ENPP1 |
| F9 | Coagulation Factor IX | Protein Coding | GC0XP139530 | 16.41922 | https://www.genecards.org/cgi-bin/carddisp.pl?gene=F9 |
| MYH11 | Myosin Heavy Chain 11 | Protein Coding | GC16M015704 | 16.36777 | https://www.genecards.org/cgi-bin/carddisp.pl?gene=MYH11 |
| F12 | Coagulation Factor XII | Protein Coding | GC05M177402 | 16.31722 | https://www.genecards.org/cgi-bin/carddisp.pl?gene=F12 |
| TGFB2 | Transforming Growth Factor Beta 2 | Protein Coding | GC01P218345 | 16.30956 | https://www.genecards.org/cgi-bin/carddisp.pl?gene=TGFB2 |
| TNFRSF1A | TNF Receptor Superfamily Member 1A | Protein Coding | GC12M006328 | 16.30193 | https://www.genecards.org/cgi-bin/carddisp.pl?gene=TNFRSF1A |
| LEP | Leptin | Protein Coding | GC07P128241 | 16.26853 | https://www.genecards.org/cgi-bin/carddisp.pl?gene=LEP |
| ACVRL1 | Activin A Receptor Like Type 1 | Protein Coding | GC12P051906 | 16.26799 | https://www.genecards.org/cgi-bin/carddisp.pl?gene=ACVRL1 |
| F8 | Coagulation Factor VIII | Protein Coding | GC0XM154835 | 16.22279 | https://www.genecards.org/cgi-bin/carddisp.pl?gene=F8 |
| HSPA1A | Heat Shock Protein Family A (Hsp70) Member 1A | Protein Coding | GC06P049370 | 16.18708 | https://www.genecards.org/cgi-bin/carddisp.pl?gene=HSPA1A |
| MBP | Myelin Basic Protein | Protein Coding | GC18M076978 | 16.1468 | https://www.genecards.org/cgi-bin/carddisp.pl?gene=MBP |
| NOS1 | Nitric Oxide Synthase 1 | Protein Coding | GC12M117208 | 16.08305 | https://www.genecards.org/cgi-bin/carddisp.pl?gene=NOS1 |
| MPO | Myeloperoxidase | Protein Coding | GC17M058269 | 16.07304 | https://www.genecards.org/cgi-bin/carddisp.pl?gene=MPO |
| ABCC6 | ATP Binding Cassette Subfamily C Member 6 | Protein Coding | GC16M016148 | 15.97442 | https://www.genecards.org/cgi-bin/carddisp.pl?gene=ABCC6 |
| SMAD3 | SMAD Family Member 3 | Protein Coding | GC15P067063 | 15.96178 | https://www.genecards.org/cgi-bin/carddisp.pl?gene=SMAD3 |
| SH2B3 | SH2B Adaptor Protein 3 | Protein Coding | GC12P111405 | 15.78274 | https://www.genecards.org/cgi-bin/carddisp.pl?gene=SH2B3 |
| CCL3 | C-C Motif Chemokine Ligand 3 | Protein Coding | GC17M036088 | 15.73443 | https://www.genecards.org/cgi-bin/carddisp.pl?gene=CCL3 |
| KCNJ5 | Potassium Inwardly Rectifying Channel Subfamily J Member 5 | Protein Coding | GC11P128891 | 15.70919 | https://www.genecards.org/cgi-bin/carddisp.pl?gene=KCNJ5 |
| RETN | Resistin | Protein Coding | GC19P007669 | 15.66459 | https://www.genecards.org/cgi-bin/carddisp.pl?gene=RETN |
| TNFRSF11B | TNF Receptor Superfamily Member 11b | Protein Coding | GC08M118923 | 15.63101 | https://www.genecards.org/cgi-bin/carddisp.pl?gene=TNFRSF11B |
| ANXA5 | Annexin A5 | Protein Coding | GC04M121667 | 15.58912 | https://www.genecards.org/cgi-bin/carddisp.pl?gene=ANXA5 |
| NPY | Neuropeptide Y | Protein Coding | GC07P024290 | 15.5726 | https://www.genecards.org/cgi-bin/carddisp.pl?gene=NPY |
| CASP1 | Caspase 1 | Protein Coding | GC11M105025 | 15.56023 | https://www.genecards.org/cgi-bin/carddisp.pl?gene=CASP1 |
| SERPINF2 | Serpin Family F Member 2 | Protein Coding | GC17P001742 | 15.53868 | https://www.genecards.org/cgi-bin/carddisp.pl?gene=SERPINF2 |
| AOC3 | Amine Oxidase Copper Containing 3 | Protein Coding | GC17P042851 | 15.38074 | https://www.genecards.org/cgi-bin/carddisp.pl?gene=AOC3 |
| TSPO | Translocator Protein | Protein Coding | GC22P043151 | 15.37689 | https://www.genecards.org/cgi-bin/carddisp.pl?gene=TSPO |
| EPOR | Erythropoietin Receptor | Protein Coding | GC19M011377 | 15.37292 | https://www.genecards.org/cgi-bin/carddisp.pl?gene=EPOR |
| APOA5 | Apolipoprotein A5 | Protein Coding | GC11M116789 | 15.36966 | https://www.genecards.org/cgi-bin/carddisp.pl?gene=APOA5 |
| TGFBR2 | Transforming Growth Factor Beta Receptor 2 | Protein Coding | GC03P030623 | 15.35703 | https://www.genecards.org/cgi-bin/carddisp.pl?gene=TGFBR2 |
| KCNQ1 | Potassium Voltage-Gated Channel Subfamily Q Member 1 | Protein Coding | GC11P002444 | 15.30208 | https://www.genecards.org/cgi-bin/carddisp.pl?gene=KCNQ1 |
| GDNF | Glial Cell Derived Neurotrophic Factor | Protein Coding | GC05M037812 | 15.27536 | https://www.genecards.org/cgi-bin/carddisp.pl?gene=GDNF |
| TBXA2R | Thromboxane A2 Receptor | Protein Coding | GC19M003594 | 15.24187 | https://www.genecards.org/cgi-bin/carddisp.pl?gene=TBXA2R |
| PSEN1 | Presenilin 1 | Protein Coding | GC14P073136 | 15.23721 | https://www.genecards.org/cgi-bin/carddisp.pl?gene=PSEN1 |
| FGG | Fibrinogen Gamma Chain | Protein Coding | GC04M154604 | 15.18859 | https://www.genecards.org/cgi-bin/carddisp.pl?gene=FGG |
| HDAC9 | Histone Deacetylase 9 | Protein Coding | GC07P018086 | 15.16603 | https://www.genecards.org/cgi-bin/carddisp.pl?gene=HDAC9 |
| LIPC | Lipase C, Hepatic Type | Protein Coding | GC15P058410 | 15.14464 | https://www.genecards.org/cgi-bin/carddisp.pl?gene=LIPC |
| ADRB1 | Adrenoceptor Beta 1 | Protein Coding | GC10P114044 | 15.11231 | https://www.genecards.org/cgi-bin/carddisp.pl?gene=ADRB1 |
| ALDH2 | Aldehyde Dehydrogenase 2 Family Member | Protein Coding | GC12P111766 | 15.03029 | https://www.genecards.org/cgi-bin/carddisp.pl?gene=ALDH2 |
| AGER | Advanced Glycosylation End-Product Specific Receptor | Protein Coding | GC06M032180 | 14.93307 | https://www.genecards.org/cgi-bin/carddisp.pl?gene=AGER |
| MYLK | Myosin Light Chain Kinase | Protein Coding | GC03M123610 | 14.92064 | https://www.genecards.org/cgi-bin/carddisp.pl?gene=MYLK |
| HBA1 | Hemoglobin Subunit Alpha 1 | Protein Coding | GC16P001557 | 14.91808 | https://www.genecards.org/cgi-bin/carddisp.pl?gene=HBA1 |
| ENTPD1 | Ectonucleoside Triphosphate Diphosphohydrolase 1 | Protein Coding | GC10P095711 | 14.89822 | https://www.genecards.org/cgi-bin/carddisp.pl?gene=ENTPD1 |
| BMP7 | Bone Morphogenetic Protein 7 | Protein Coding | GC20M057168 | 14.74184 | https://www.genecards.org/cgi-bin/carddisp.pl?gene=BMP7 |
| APOC3 | Apolipoprotein C3 | Protein Coding | GC11P116829 | 14.72394 | https://www.genecards.org/cgi-bin/carddisp.pl?gene=APOC3 |
| GATA4 | GATA Binding Protein 4 | Protein Coding | GC08P011676 | 14.71034 | https://www.genecards.org/cgi-bin/carddisp.pl?gene=GATA4 |
| SLC6A4 | Solute Carrier Family 6 Member 4 | Protein Coding | GC17M030194 | 14.70601 | https://www.genecards.org/cgi-bin/carddisp.pl?gene=SLC6A4 |
| MPL | MPL Proto-Oncogene, Thrombopoietin Receptor | Protein Coding | GC01P043337 | 14.56329 | https://www.genecards.org/cgi-bin/carddisp.pl?gene=MPL |
| HP | Haptoglobin | Protein Coding | GC16P072089 | 14.55521 | https://www.genecards.org/cgi-bin/carddisp.pl?gene=HP |
| SIRT1 | Sirtuin 1 | Protein Coding | GC10P067884 | 14.46672 | https://www.genecards.org/cgi-bin/carddisp.pl?gene=SIRT1 |
| SLC2A10 | Solute Carrier Family 2 Member 10 | Protein Coding | GC20P046709 | 14.43497 | https://www.genecards.org/cgi-bin/carddisp.pl?gene=SLC2A10 |
| THPO | Thrombopoietin | Protein Coding | GC03M184371 | 14.39805 | https://www.genecards.org/cgi-bin/carddisp.pl?gene=THPO |
| SMAD4 | SMAD Family Member 4 | Protein Coding | GC18P051028 | 14.24477 | https://www.genecards.org/cgi-bin/carddisp.pl?gene=SMAD4 |
| AGTR2 | Angiotensin II Receptor Type 2 | Protein Coding | GC0XP116170 | 14.16125 | https://www.genecards.org/cgi-bin/carddisp.pl?gene=AGTR2 |
| ADORA2A | Adenosine A2a Receptor | Protein Coding | GC22P024417 | 14.12571 | https://www.genecards.org/cgi-bin/carddisp.pl?gene=ADORA2A |
| NINJ2 | Ninjurin 2 | Protein Coding | GC12M000564 | 14.07181 | https://www.genecards.org/cgi-bin/carddisp.pl?gene=NINJ2 |
| CPB2 | Carboxypeptidase B2 | Protein Coding | GC13M046053 | 14.05422 | https://www.genecards.org/cgi-bin/carddisp.pl?gene=CPB2 |
| NFE2L2 | Nuclear Factor, Erythroid 2 Like 2 | Protein Coding | GC02M177227 | 14.01657 | https://www.genecards.org/cgi-bin/carddisp.pl?gene=NFE2L2 |
| TGFBR1 | Transforming Growth Factor Beta Receptor 1 | Protein Coding | GC09P099104 | 14.01531 | https://www.genecards.org/cgi-bin/carddisp.pl?gene=TGFBR1 |
| ADORA3 | Adenosine A3 Receptor | Protein Coding | GC01M111499 | 13.99902 | https://www.genecards.org/cgi-bin/carddisp.pl?gene=ADORA3 |
| CYP11B2 | Cytochrome P450 Family 11 Subfamily B Member 2 | Protein Coding | GC08M142910 | 13.96401 | https://www.genecards.org/cgi-bin/carddisp.pl?gene=CYP11B2 |
| F11 | Coagulation Factor XI | Protein Coding | GC04P186265 | 13.8871 | https://www.genecards.org/cgi-bin/carddisp.pl?gene=F11 |
| KDR | Kinase Insert Domain Receptor | Protein Coding | GC04M055078 | 13.87503 | https://www.genecards.org/cgi-bin/carddisp.pl?gene=KDR |
| XDH | Xanthine Dehydrogenase | Protein Coding | GC02M031334 | 13.87344 | https://www.genecards.org/cgi-bin/carddisp.pl?gene=XDH |
| CP | Ceruloplasmin | Protein Coding | GC03M149162 | 13.69438 | https://www.genecards.org/cgi-bin/carddisp.pl?gene=CP |
| CDKN2A | Cyclin Dependent Kinase Inhibitor 2A | Protein Coding | GC09M021967 | 13.68267 | https://www.genecards.org/cgi-bin/carddisp.pl?gene=CDKN2A |
| PDE5A | Phosphodiesterase 5A | Protein Coding | GC04M119494 | 13.67711 | https://www.genecards.org/cgi-bin/carddisp.pl?gene=PDE5A |
| PCSK9 | Proprotein Convertase Subtilisin/Kexin Type 9 | Protein Coding | GC01P055039 | 13.67225 | https://www.genecards.org/cgi-bin/carddisp.pl?gene=PCSK9 |
| CAT | Catalase | Protein Coding | GC11P034460 | 13.64813 | https://www.genecards.org/cgi-bin/carddisp.pl?gene=CAT |
| LTC4S | Leukotriene C4 Synthase | Protein Coding | GC05P179793 | 13.60776 | https://www.genecards.org/cgi-bin/carddisp.pl?gene=LTC4S |
| CCR5 | C-C Motif Chemokine Receptor 5 | Protein Coding | GC03P046383 | 13.59129 | https://www.genecards.org/cgi-bin/carddisp.pl?gene=CCR5 |
| CALCA | Calcitonin Related Polypeptide Alpha | Protein Coding | GC11M014945 | 13.46399 | https://www.genecards.org/cgi-bin/carddisp.pl?gene=CALCA |
| MAPK3 | Mitogen-Activated Protein Kinase 3 | Protein Coding | GC16M030117 | 13.45727 | https://www.genecards.org/cgi-bin/carddisp.pl?gene=MAPK3 |
| CYP3A5 | Cytochrome P450 Family 3 Subfamily A Member 5 | Protein Coding | GC07M099648 | 13.45368 | https://www.genecards.org/cgi-bin/carddisp.pl?gene=CYP3A5 |
| CALR | Calreticulin | Protein Coding | GC19P012938 | 13.43088 | https://www.genecards.org/cgi-bin/carddisp.pl?gene=CALR |
| CYP2C9 | Cytochrome P450 Family 2 Subfamily C Member 9 | Protein Coding | GC10P094938 | 13.40577 | https://www.genecards.org/cgi-bin/carddisp.pl?gene=CYP2C9 |
| PARP1 | Poly(ADP-Ribose) Polymerase 1 | Protein Coding | GC01M226360 | 13.29249 | https://www.genecards.org/cgi-bin/carddisp.pl?gene=PARP1 |
| NGF | Nerve Growth Factor | Protein Coding | GC01M115285 | 13.17869 | https://www.genecards.org/cgi-bin/carddisp.pl?gene=NGF |
| MT-CYB | Mitochondrially Encoded Cytochrome B | Protein Coding | GCMTP014749 | 13.17793 | https://www.genecards.org/cgi-bin/carddisp.pl?gene=MT-CYB |
| TNFRSF12A | TNF Receptor Superfamily Member 12A | Protein Coding | GC16P003018 | 13.15642 | https://www.genecards.org/cgi-bin/carddisp.pl?gene=TNFRSF12A |
| G6PD | Glucose-6-Phosphate Dehydrogenase | Protein Coding | GC0XM154531 | 13.09201 | https://www.genecards.org/cgi-bin/carddisp.pl?gene=G6PD |
| CYBA | Cytochrome B-245 Alpha Chain | Protein Coding | GC16M088643 | 13.08238 | https://www.genecards.org/cgi-bin/carddisp.pl?gene=CYBA |
| DCX | Doublecortin | Protein Coding | GC0XM111293 | 13.07258 | https://www.genecards.org/cgi-bin/carddisp.pl?gene=DCX |
| PPARA | Peroxisome Proliferator Activated Receptor Alpha | Protein Coding | GC22P046150 | 13.06615 | https://www.genecards.org/cgi-bin/carddisp.pl?gene=PPARA |
| C3 | Complement C3 | Protein Coding | GC19M006677 | 13.02614 | https://www.genecards.org/cgi-bin/carddisp.pl?gene=C3 |
| CYP4F2 | Cytochrome P450 Family 4 Subfamily F Member 2 | Protein Coding | GC19M015878 | 13.01329 | https://www.genecards.org/cgi-bin/carddisp.pl?gene=CYP4F2 |
| GPT | Glutamic--Pyruvic Transaminase | Protein Coding | GC08P144502 | 12.99694 | https://www.genecards.org/cgi-bin/carddisp.pl?gene=GPT |
| CSF3 | Colony Stimulating Factor 3 | Protein Coding | GC17P040015 | 12.99441 | https://www.genecards.org/cgi-bin/carddisp.pl?gene=CSF3 |
| CCR2 | C-C Motif Chemokine Receptor 2 | Protein Coding | GC03P046356 | 12.9136 | https://www.genecards.org/cgi-bin/carddisp.pl?gene=CCR2 |
| PITX2 | Paired Like Homeodomain 2 | Protein Coding | GC04M110617 | 12.91306 | https://www.genecards.org/cgi-bin/carddisp.pl?gene=PITX2 |
| NKX2-5 | NK2 Homeobox 5 | Protein Coding | GC05M173232 | 12.90134 | https://www.genecards.org/cgi-bin/carddisp.pl?gene=NKX2-5 |
| PROC | Protein C, Inactivator Of Coagulation Factors Va And VIIIa | Protein Coding | GC02P127418 | 12.81688 | https://www.genecards.org/cgi-bin/carddisp.pl?gene=PROC |
| MMP1 | Matrix Metallopeptidase 1 | Protein Coding | GC11M102810 | 12.74587 | https://www.genecards.org/cgi-bin/carddisp.pl?gene=MMP1 |
| HBB | Hemoglobin Subunit Beta | Protein Coding | GC11M005377 | 12.63474 | https://www.genecards.org/cgi-bin/carddisp.pl?gene=HBB |
| CYP2J2 | Cytochrome P450 Family 2 Subfamily J Member 2 | Protein Coding | GC01M059893 | 12.52341 | https://www.genecards.org/cgi-bin/carddisp.pl?gene=CYP2J2 |
| TET2 | Tet Methylcytosine Dioxygenase 2 | Protein Coding | GC04P105145 | 12.50866 | https://www.genecards.org/cgi-bin/carddisp.pl?gene=TET2 |
| VDR | Vitamin D Receptor | Protein Coding | GC12M047841 | 12.50705 | https://www.genecards.org/cgi-bin/carddisp.pl?gene=VDR |
| TF | Transferrin | Protein Coding | GC03P133666 | 12.47273 | https://www.genecards.org/cgi-bin/carddisp.pl?gene=TF |
| ECE1 | Endothelin Converting Enzyme 1 | Protein Coding | GC01M021217 | 12.42215 | https://www.genecards.org/cgi-bin/carddisp.pl?gene=ECE1 |
| ABCB1 | ATP Binding Cassette Subfamily B Member 1 | Protein Coding | GC07M087504 | 12.32243 | https://www.genecards.org/cgi-bin/carddisp.pl?gene=ABCB1 |
| ACTC1 | Actin Alpha Cardiac Muscle 1 | Protein Coding | GC15M034788 | 12.32138 | https://www.genecards.org/cgi-bin/carddisp.pl?gene=ACTC1 |
| BACE1 | Beta-Secretase 1 | Protein Coding | GC11M117285 | 12.12954 | https://www.genecards.org/cgi-bin/carddisp.pl?gene=BACE1 |
| GDF15 | Growth Differentiation Factor 15 | Protein Coding | GC19P024469 | 12.12225 | https://www.genecards.org/cgi-bin/carddisp.pl?gene=GDF15 |
| SCN5A | Sodium Voltage-Gated Channel Alpha Subunit 5 | Protein Coding | GC03M038549 | 12.11913 | https://www.genecards.org/cgi-bin/carddisp.pl?gene=SCN5A |
| HFE | Homeostatic Iron Regulator | Protein Coding | GC06P026087 | 12.04772 | https://www.genecards.org/cgi-bin/carddisp.pl?gene=HFE |
| CDKN2B | Cyclin Dependent Kinase Inhibitor 2B | Protein Coding | GC09M022002 | 11.95665 | https://www.genecards.org/cgi-bin/carddisp.pl?gene=CDKN2B |
| MYBPC3 | Myosin Binding Protein C3 | Protein Coding | GC11M063608 | 11.93822 | https://www.genecards.org/cgi-bin/carddisp.pl?gene=MYBPC3 |
| KL | Klotho | Protein Coding | GC13P033016 | 11.93746 | https://www.genecards.org/cgi-bin/carddisp.pl?gene=KL |
| NOTCH1 | Notch Receptor 1 | Protein Coding | GC09M136623 | 11.92902 | https://www.genecards.org/cgi-bin/carddisp.pl?gene=NOTCH1 |
| CCL11 | C-C Motif Chemokine Ligand 11 | Protein Coding | GC17P034285 | 11.86037 | https://www.genecards.org/cgi-bin/carddisp.pl?gene=CCL11 |
| SERPINA1 | Serpin Family A Member 1 | Protein Coding | GC14M094376 | 11.84324 | https://www.genecards.org/cgi-bin/carddisp.pl?gene=SERPINA1 |
| BCL2 | BCL2 Apoptosis Regulator | Protein Coding | GC18M063123 | 11.83012 | https://www.genecards.org/cgi-bin/carddisp.pl?gene=BCL2 |
| POLG | DNA Polymerase Gamma, Catalytic Subunit | Protein Coding | GC15M089313 | 11.82826 | https://www.genecards.org/cgi-bin/carddisp.pl?gene=POLG |
| UCP2 | Uncoupling Protein 2 | Protein Coding | GC11M073974 | 11.81461 | https://www.genecards.org/cgi-bin/carddisp.pl?gene=UCP2 |
| ACTB | Actin Beta | Protein Coding | GC07M005527 | 11.79184 | https://www.genecards.org/cgi-bin/carddisp.pl?gene=ACTB |
| TREX1 | Three Prime Repair Exonuclease 1 | Protein Coding | GC03P048466 | 11.74746 | https://www.genecards.org/cgi-bin/carddisp.pl?gene=TREX1 |
| CASP9 | Caspase 9 | Protein Coding | GC01M015491 | 11.74602 | https://www.genecards.org/cgi-bin/carddisp.pl?gene=CASP9 |
| ZFHX3 | Zinc Finger Homeobox 3 | Protein Coding | GC16M072782 | 11.72605 | https://www.genecards.org/cgi-bin/carddisp.pl?gene=ZFHX3 |
| PRNP | Prion Protein | Protein Coding | GC20P004686 | 11.63942 | https://www.genecards.org/cgi-bin/carddisp.pl?gene=PRNP |
| GCKR | Glucokinase Regulator | Protein Coding | GC02P027496 | 11.63197 | https://www.genecards.org/cgi-bin/carddisp.pl?gene=GCKR |
| MYH7 | Myosin Heavy Chain 7 | Protein Coding | GC14M023412 | 11.62733 | https://www.genecards.org/cgi-bin/carddisp.pl?gene=MYH7 |
| IL4R | Interleukin 4 Receptor | Protein Coding | GC16P027325 | 11.61339 | https://www.genecards.org/cgi-bin/carddisp.pl?gene=IL4R |
| CSF2 | Colony Stimulating Factor 2 | Protein Coding | GC05P132073 | 11.61264 | https://www.genecards.org/cgi-bin/carddisp.pl?gene=CSF2 |
| SELL | Selectin L | Protein Coding | GC01M169690 | 11.54739 | https://www.genecards.org/cgi-bin/carddisp.pl?gene=SELL |
| PRL | Prolactin | Protein Coding | GC06M022287 | 11.51867 | https://www.genecards.org/cgi-bin/carddisp.pl?gene=PRL |
| CTLA4 | Cytotoxic T-Lymphocyte Associated Protein 4 | Protein Coding | GC02P203867 | 11.50851 | https://www.genecards.org/cgi-bin/carddisp.pl?gene=CTLA4 |
| NLRP3 | NLR Family Pyrin Domain Containing 3 | Protein Coding | GC01P247415 | 11.4895 | https://www.genecards.org/cgi-bin/carddisp.pl?gene=NLRP3 |
| TGFB3 | Transforming Growth Factor Beta 3 | Protein Coding | GC14M075958 | 11.47956 | https://www.genecards.org/cgi-bin/carddisp.pl?gene=TGFB3 |
| AMPD1 | Adenosine Monophosphate Deaminase 1 | Protein Coding | GC01M114673 | 11.44257 | https://www.genecards.org/cgi-bin/carddisp.pl?gene=AMPD1 |
| MTR | 5-Methyltetrahydrofolate-Homocysteine Methyltransferase | Protein Coding | GC01P236795 | 11.40136 | https://www.genecards.org/cgi-bin/carddisp.pl?gene=MTR |
| FLT1 | Fms Related Receptor Tyrosine Kinase 1 | Protein Coding | GC13M028300 | 11.38612 | https://www.genecards.org/cgi-bin/carddisp.pl?gene=FLT1 |
| HLA-DRB1 | Major Histocompatibility Complex, Class II, DR Beta 1 | Protein Coding | GC06M032578 | 11.37722 | https://www.genecards.org/cgi-bin/carddisp.pl?gene=HLA-DRB1 |
| CD40 | CD40 Molecule | Protein Coding | GC20P046118 | 11.36295 | https://www.genecards.org/cgi-bin/carddisp.pl?gene=CD40 |
| MAPK14 | Mitogen-Activated Protein Kinase 14 | Protein Coding | GC06P049507 | 11.35216 | https://www.genecards.org/cgi-bin/carddisp.pl?gene=MAPK14 |
| TIMP1 | TIMP Metallopeptidase Inhibitor 1 | Protein Coding | GC0XP047583 | 11.34628 | https://www.genecards.org/cgi-bin/carddisp.pl?gene=TIMP1 |
| IL1R1 | Interleukin 1 Receptor Type 1 | Protein Coding | GC02P102136 | 11.31005 | https://www.genecards.org/cgi-bin/carddisp.pl?gene=IL1R1 |
| ITGB2 | Integrin Subunit Beta 2 | Protein Coding | GC21M044885 | 11.24879 | https://www.genecards.org/cgi-bin/carddisp.pl?gene=ITGB2 |
| ITGB1 | Integrin Subunit Beta 1 | Protein Coding | GC10M032890 | 11.2369 | https://www.genecards.org/cgi-bin/carddisp.pl?gene=ITGB1 |
| ACE2 | Angiotensin Converting Enzyme 2 | Protein Coding | GC0XM015494 | 11.22502 | https://www.genecards.org/cgi-bin/carddisp.pl?gene=ACE2 |
| GSTM1 | Glutathione S-Transferase Mu 1 | Protein Coding | GC01P109687 | 11.20813 | https://www.genecards.org/cgi-bin/carddisp.pl?gene=GSTM1 |
| PKD1 | Polycystin 1, Transient Receptor Potential Channel Interacting | Protein Coding | GC16M002394 | 11.16691 | https://www.genecards.org/cgi-bin/carddisp.pl?gene=PKD1 |
| MBL2 | Mannose Binding Lectin 2 | Protein Coding | GC10M052760 | 11.14588 | https://www.genecards.org/cgi-bin/carddisp.pl?gene=MBL2 |
| C5 | Complement C5 | Protein Coding | GC09M120952 | 11.13597 | https://www.genecards.org/cgi-bin/carddisp.pl?gene=C5 |
| NTF3 | Neurotrophin 3 | Protein Coding | GC12P005432 | 11.1251 | https://www.genecards.org/cgi-bin/carddisp.pl?gene=NTF3 |
| MFAP5 | Microfibril Associated Protein 5 | Protein Coding | GC12M008637 | 11.10809 | https://www.genecards.org/cgi-bin/carddisp.pl?gene=MFAP5 |
| KRIT1 | KRIT1 Ankyrin Repeat Containing | Protein Coding | GC07M092198 | 11.09691 | https://www.genecards.org/cgi-bin/carddisp.pl?gene=KRIT1 |
| COG2 | Component Of Oligomeric Golgi Complex 2 | Protein Coding | GC01P230642 | 11.09378 | https://www.genecards.org/cgi-bin/carddisp.pl?gene=COG2 |
| TBXAS1 | Thromboxane A Synthase 1 | Protein Coding | GC07P139777 | 11.00373 | https://www.genecards.org/cgi-bin/carddisp.pl?gene=TBXAS1 |
| ABO | ABO, Alpha 1-3-N-Acetylgalactosaminyltransferase And Alpha 1-3-Galactosyltransferase | Protein Coding | GC09M133250 | 10.90793 | https://www.genecards.org/cgi-bin/carddisp.pl?gene=ABO |
| MIF | Macrophage Migration Inhibitory Factor | Protein Coding | GC22P023894 | 10.90735 | https://www.genecards.org/cgi-bin/carddisp.pl?gene=MIF |
| MAPK1 | Mitogen-Activated Protein Kinase 1 | Protein Coding | GC22M021754 | 10.88578 | https://www.genecards.org/cgi-bin/carddisp.pl?gene=MAPK1 |
| SERPIND1 | Serpin Family D Member 1 | Protein Coding | GC22P020868 | 10.86389 | https://www.genecards.org/cgi-bin/carddisp.pl?gene=SERPIND1 |
| AIF1 | Allograft Inflammatory Factor 1 | Protein Coding | GC06P049351 | 10.84156 | https://www.genecards.org/cgi-bin/carddisp.pl?gene=AIF1 |
| MYH6 | Myosin Heavy Chain 6 | Protein Coding | GC14M023380 | 10.82133 | https://www.genecards.org/cgi-bin/carddisp.pl?gene=MYH6 |
| LGALS3 | Galectin 3 | Protein Coding | GC14P055124 | 10.76095 | https://www.genecards.org/cgi-bin/carddisp.pl?gene=LGALS3 |
| IGFBP3 | Insulin Like Growth Factor Binding Protein 3 | Protein Coding | GC07M045912 | 10.75681 | https://www.genecards.org/cgi-bin/carddisp.pl?gene=IGFBP3 |
| MMP12 | Matrix Metallopeptidase 12 | Protein Coding | GC11M102862 | 10.67837 | https://www.genecards.org/cgi-bin/carddisp.pl?gene=MMP12 |
| CDK5 | Cyclin Dependent Kinase 5 | Protein Coding | GC07M151053 | 10.65998 | https://www.genecards.org/cgi-bin/carddisp.pl?gene=CDK5 |
| MGP | Matrix Gla Protein | Protein Coding | GC12M014985 | 10.60416 | https://www.genecards.org/cgi-bin/carddisp.pl?gene=MGP |
| PTX3 | Pentraxin 3 | Protein Coding | GC03P157436 | 10.57813 | https://www.genecards.org/cgi-bin/carddisp.pl?gene=PTX3 |
| CYP4A11 | Cytochrome P450 Family 4 Subfamily A Member 11 | Protein Coding | GC01M046929 | 10.57688 | https://www.genecards.org/cgi-bin/carddisp.pl?gene=CYP4A11 |
| DES | Desmin | Protein Coding | GC02P219418 | 10.54569 | https://www.genecards.org/cgi-bin/carddisp.pl?gene=DES |
| GJA4 | Gap Junction Protein Alpha 4 | Protein Coding | GC01P034792 | 10.53476 | https://www.genecards.org/cgi-bin/carddisp.pl?gene=GJA4 |
| FCGR2A | Fc Fragment Of IgG Receptor IIa | Protein Coding | GC01P161505 | 10.51714 | https://www.genecards.org/cgi-bin/carddisp.pl?gene=FCGR2A |
| FOS | Fos Proto-Oncogene, AP-1 Transcription Factor Subunit | Protein Coding | GC14P075278 | 10.51372 | https://www.genecards.org/cgi-bin/carddisp.pl?gene=FOS |
| IL13 | Interleukin 13 | Protein Coding | GC05P132656 | 10.49295 | https://www.genecards.org/cgi-bin/carddisp.pl?gene=IL13 |
| IL17A | Interleukin 17A | Protein Coding | GC06P052186 | 10.4522 | https://www.genecards.org/cgi-bin/carddisp.pl?gene=IL17A |
| SLC9A1 | Solute Carrier Family 9 Member A1 | Protein Coding | GC01M027109 | 10.45126 | https://www.genecards.org/cgi-bin/carddisp.pl?gene=SLC9A1 |
| PTEN | Phosphatase And Tensin Homolog | Protein Coding | GC10P087863 | 10.44431 | https://www.genecards.org/cgi-bin/carddisp.pl?gene=PTEN |
| MAT2A | Methionine Adenosyltransferase 2A | Protein Coding | GC02P085538 | 10.44302 | https://www.genecards.org/cgi-bin/carddisp.pl?gene=MAT2A |
| CD34 | CD34 Molecule | Protein Coding | GC01M207880 | 10.44272 | https://www.genecards.org/cgi-bin/carddisp.pl?gene=CD34 |
| SELPLG | Selectin P Ligand | Protein Coding | GC12M108621 | 10.39463 | https://www.genecards.org/cgi-bin/carddisp.pl?gene=SELPLG |
| ANGPT2 | Angiopoietin 2 | Protein Coding | GC08M006499 | 10.38176 | https://www.genecards.org/cgi-bin/carddisp.pl?gene=ANGPT2 |
| FOXE3 | Forkhead Box E3 | Protein Coding | GC01P047416 | 10.34152 | https://www.genecards.org/cgi-bin/carddisp.pl?gene=FOXE3 |
| HTR2A | 5-Hydroxytryptamine Receptor 2A | Protein Coding | GC13M046831 | 10.31113 | https://www.genecards.org/cgi-bin/carddisp.pl?gene=HTR2A |
| RPS27A | Ribosomal Protein S27a | Protein Coding | GC02P055231 | 10.23181 | https://www.genecards.org/cgi-bin/carddisp.pl?gene=RPS27A |
| CYP2C8 | Cytochrome P450 Family 2 Subfamily C Member 8 | Protein Coding | GC10M095038 | 10.21193 | https://www.genecards.org/cgi-bin/carddisp.pl?gene=CYP2C8 |
| NPR3 | Natriuretic Peptide Receptor 3 | Protein Coding | GC05P032689 | 10.14338 | https://www.genecards.org/cgi-bin/carddisp.pl?gene=NPR3 |
| XBP1 | X-Box Binding Protein 1 | Protein Coding | GC22M028794 | 10.13226 | https://www.genecards.org/cgi-bin/carddisp.pl?gene=XBP1 |
| CTF1 | Cardiotrophin 1 | Protein Coding | GC16P030910 | 10.08212 | https://www.genecards.org/cgi-bin/carddisp.pl?gene=CTF1 |
| IL2 | Interleukin 2 | Protein Coding | GC04M122451 | 10.03248 | https://www.genecards.org/cgi-bin/carddisp.pl?gene=IL2 |
