# Supplementary figures and images for: Construction Immune Related Feed-Forward Loop Network Reveals Angiotensin II Receptor Blocker as Potential Neuroprotective Drug for Ischemic Stroke
Source: Front Genet. 2022 Mar 28;13:811571. doi: 10.3389/fgene.2022.811571 (PMC8995882; doi:10.3389/fgene.2022.811571)

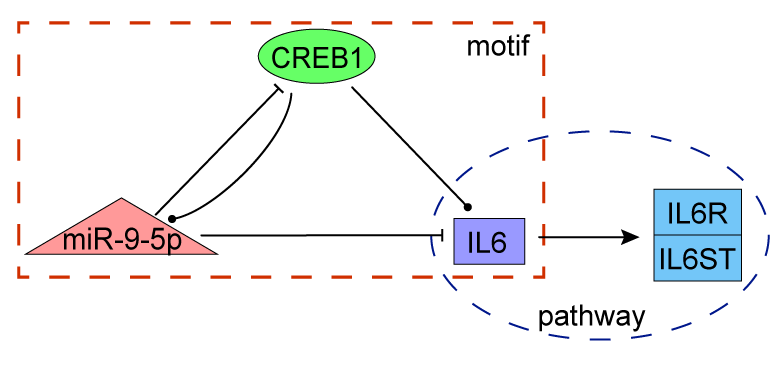

Supplement: Supplementary file 2 [file Image3.TIF]

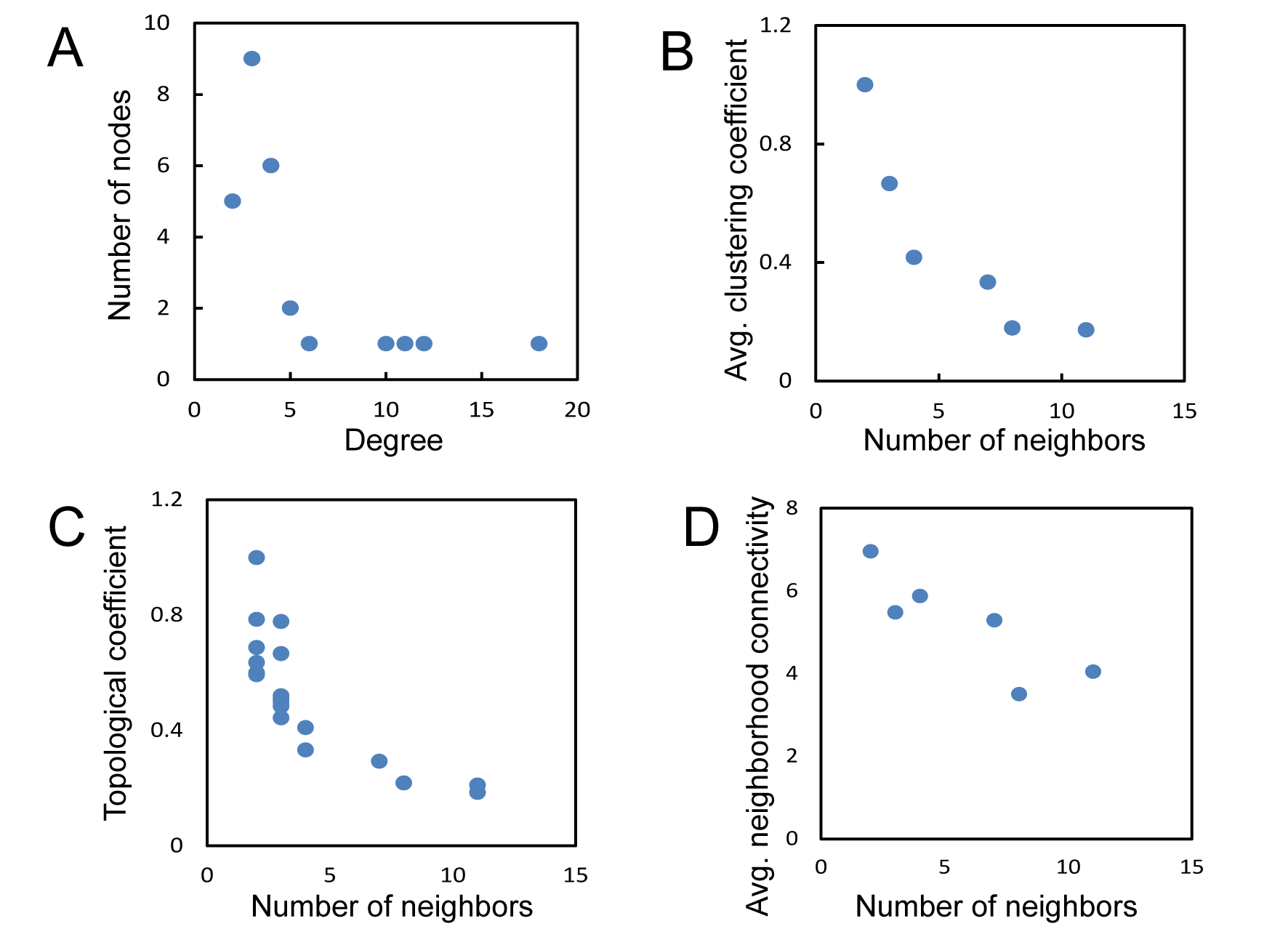

Supplement: Supplementary file 3 [file Image2.TIF]

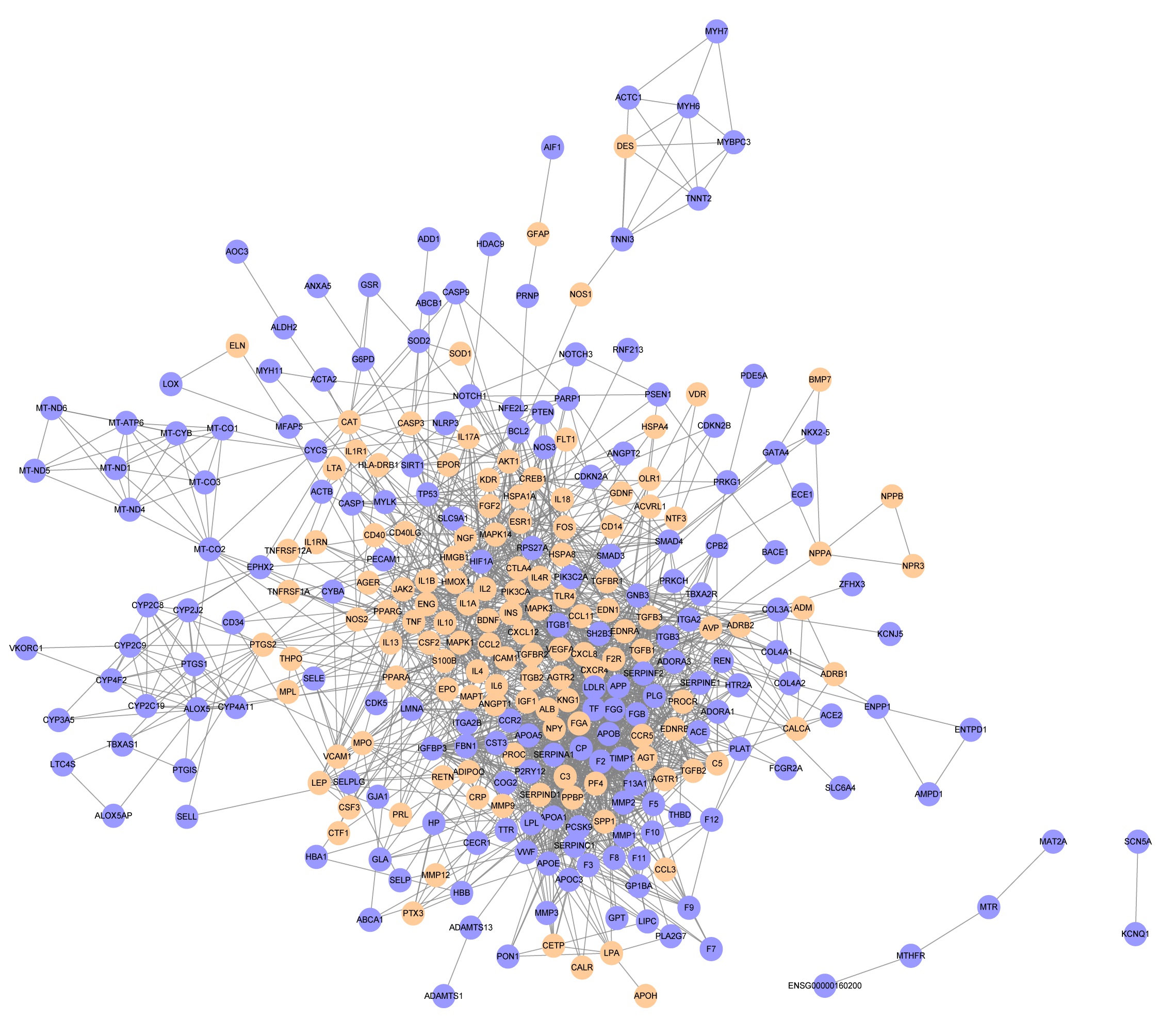

Supplement: Supplementary file 4 [file Image1.TIF]
