# Supplementary material for: Construction Immune Related Feed-Forward Loop Network Reveals Angiotensin II Receptor Blocker as Potential Neuroprotective Drug for Ischemic Stroke
Source: Front Genet. 2022 Mar 28;13:811571. doi: 10.3389/fgene.2022.811571 (PMC8995882; doi:10.3389/fgene.2022.811571)
Supplement: Supplementary file 5 [file Table2.DOCX]

Table S2 Ischemic stroke immune related genes.

| ACVRL1 | CCL11 | EPO | IL18 | MIF | PTX3 |
| --- | --- | --- | --- | --- | --- |
| ADA2 | CCL2 | EPOR | IL1A | MMP12 | RETN |
| ADIPOQ | CCL3 | ESR1 | IL1B | MMP9 | S100B |
| ADM | CCR5 | F2R | IL1R1 | MPL | SERPINA3 |
| ADRB1 | CD14 | FGA | IL1RN | MPO | SERPIND1 |
| ADRB2 | CD40 | FGF2 | IL2 | NGF | SOD1 |
| AGER | CD40LG | FLT1 | IL4 | NOS1 | SPP1 |
| AGT | CETP | FOS | IL4R | NOS2 | TGFB1 |
| AGTR1 | CREB1 | GDF15 | IL6 | NPPA | TGFB2 |
| AGTR2 | CRP | GDNF | INS | NPPB | TGFB3 |
| AKT1 | CSF2 | GFAP | ITGB2 | NPR3 | TGFBR1 |
| ALB | CSF3 | HFE | JAK2 | NPY | TGFBR2 |
| ANGPT1 | CTF1 | HLA-DRB1 | KDR | NTF3 | THPO |
| APOH | CTLA4 | HMGB1 | KL | OLR1 | TLR4 |
| AVP | CXCL12 | HMOX1 | KNG1 | PF4 | TNF |
| BDNF | CXCL8 | HSPA1A | LEP | PIK3CA | TNFRSF11B |
| BMP7 | CXCR4 | HSPA4 | LPA | PPARA | TNFRSF12A |
| C3 | DES | HSPA8 | LTA | PPARG | TNFRSF1A |
| C5 | EDN1 | ICAM1 | MAPK1 | PPBP | VCAM1 |
| CALCA | EDNRA | IGF1 | MAPK14 | PRL | VDR |
| CALR | EDNRB | IL10 | MAPK3 | PROC | VEGFA |
| CASP3 | ELN | IL13 | MAPT | PROCR |  |
| CAT | ENG | IL17A | MBL2 | PTGS2 |  |
