# Supplementary material for: Construction Immune Related Feed-Forward Loop Network Reveals Angiotensin II Receptor Blocker as Potential Neuroprotective Drug for Ischemic Stroke
Source: Front Genet. 2022 Mar 28;13:811571. doi: 10.3389/fgene.2022.811571 (PMC8995882; doi:10.3389/fgene.2022.811571)
Supplement: Supplementary file 6 [file Table5.DOCX]

**Table 51 Detail information of drug-gene pairs, drug-TF pairs and drug-TF&gene pairs.**

| Catagory | Name | Drug name |
| --- | --- | --- |
| Gene | CXCR4 | Plerixafor |
|  | FGF2 | Sucralfate |
|  | FGF2 | Pentosan Polysulfate |
|  | FGF2 | Sirolimus |
|  | IL6 | Siltuximab |
|  | VEGFA | Bevacizumab |
|  | VEGFA | Ranibizumab |
|  | VEGFA | Dalteparin |
|  | VEGFA | Aflibercept |
|  | AGTR1 | Valsartan |
|  | AGTR1 | Olmesartan |
|  | AGTR1 | Losartan |
|  | AGTR1 | Candesartan cilexetil |
|  | AGTR1 | Eprosartan |
|  | AGTR1 | Telmisartan |
|  | AGTR1 | Irbesartan |
|  | AGTR1 | Tasosartan |
|  | AGTR1 | Azilsartan medoxomil |
|  | AGTR1 | Fimasartan |
|  | AGTR1 | Angiotensin II |
| TF&gene | ESR1 | Conjugated estrogens |
|  | ESR1 | Tamoxifen |
|  | ESR1 | Raloxifene |
|  | ESR1 | Toremifene |
|  | ESR1 | Medroxyprogesterone acetate |
|  | ESR1 | Fulvestrant |
|  | ESR1 | Norgestimate |
|  | ESR1 | Estramustine |
|  | ESR1 | Tibolone |
|  | ESR1 | Fluoxymesterone |
|  | ESR1 | Trilostane |
|  | ESR1 | Ethynodiol diacetate |
|  | ESR1 | Ethinyl Estradiol |
|  | ESR1 | Estradiol |
|  | ESR1 | Polyestradiol phosphate |
|  | ESR1 | Estradiol acetate |
|  | ESR1 | Estradiol benzoate |
|  | ESR1 | Estradiol cypionate |
|  | ESR1 | Estradiol dienanthate |
|  | ESR1 | Estradiol valerate |
|  | ESR1 | Estrone sulfate |
|  | ESR1 | Synthetic Conjugated Estrogens, A |
|  | ESR1 | Synthetic Conjugated Estrogens, B |
|  | ESR1 | Bazedoxifene |
|  | ESR1 | Lasofoxifene |
|  | ESR1 | Mestranol |
|  | ESR1 | Danazol |
|  | ESR1 | Quinestrol |
|  | ESR1 | Ospemifene |
|  | ESR1 | Estrone |
|  | ESR1 | Dienestrol |
|  | ESR1 | Estriol |
|  | ESR1 | Clomifene |
|  | ESR1 | Etonogestrel |
|  | ESR1 | Desogestrel |
|  | ESR1 | Progesterone |
|  | ESR1 | Diethylstilbestrol |
|  | ESR1 | Levonorgestrel |
