# Supplementary material for: Construction Immune Related Feed-Forward Loop Network Reveals Angiotensin II Receptor Blocker as Potential Neuroprotective Drug for Ischemic Stroke
Source: Front Genet. 2022 Mar 28;13:811571. doi: 10.3389/fgene.2022.811571 (PMC8995882; doi:10.3389/fgene.2022.811571)
Supplement: Supplementary file 7 [file Table3.DOCX]

Table S3 Ischemic stroke related miRNAs.

| hsa-let-7b-5p | hsa-miR-146b-5p | hsa-miR-24-3p | hsa-miR-4306 |
| --- | --- | --- | --- |
| hsa-let-7c-5p | hsa-miR-148a-3p | hsa-miR-25-3p | hsa-miR-432-5p |
| hsa-let-7e-5p | hsa-miR-148b-3p | hsa-miR-26b-5p | hsa-miR-4429 |
| hsa-let-7i-5p | hsa-miR-149-5p | hsa-miR-27a-3p | hsa-miR-4437 |
| hsa-miR-106b-5p | hsa-miR-150-5p | hsa-miR-27b-3p | hsa-miR-451a |
| hsa-miR-107 | hsa-miR-151a-3p | hsa-miR-29b-3p | hsa-miR-4656 |
| hsa-miR-10a-5p | hsa-miR-151b | hsa-miR-300 | hsa-miR-4739 |
| hsa-miR-122-5p | hsa-miR-155-5p | hsa-miR-30a-5p | hsa-miR-487b-3p |
| hsa-miR-124-3p | hsa-miR-15a-5p | hsa-miR-30d-5p | hsa-miR-487b-5p |
| hsa-miR-1246 | hsa-miR-16-5p | hsa-miR-3149 | hsa-miR-491-5p |
| hsa-miR-125a-5p | hsa-miR-17-5p | hsa-miR-3161 | hsa-miR-494-5p |
| hsa-miR-125b-2-3p | hsa-miR-181a-5p | hsa-miR-320a-3p | hsa-miR-494-3p |
| hsa-miR-125b-5p | hsa-miR-181b-5p | hsa-miR-320a-5p | hsa-miR-498-3p |
| hsa-miR-126-3p | hsa-miR-182-5p | hsa-miR-320d | hsa-miR-498-5p |
| hsa-miR-127-3p | hsa-miR-185-5p | hsa-miR-320e | hsa-miR-499a-5p |
| hsa-miR-1275 | hsa-miR-18b-5p | hsa-miR-32-3p | hsa-miR-503-5p |
| hsa-miR-128-3p | hsa-miR-1913 | hsa-miR-340-5p | hsa-miR-518b |
| hsa-miR-1299 | hsa-miR-195-5p | hsa-miR-342-3p | hsa-miR-532-5p |
| hsa-miR-130a-3p | hsa-miR-19a-3p | hsa-miR-34b-3p | hsa-miR-574-5p |
| hsa-miR-132-3p | hsa-miR-200b-3p | hsa-miR-34c-5p | hsa-miR-599 |
| hsa-miR-134-3p | hsa-miR-20a-5p | hsa-miR-361-5p | hsa-miR-616-5p |
| hsa-miR-134-5p | hsa-miR-21-5p | hsa-miR-363-3p | hsa-miR-874-3p |
| hsa-miR-135b-5p | hsa-miR-210-3p | hsa-miR-374b-5p | hsa-miR-874-5p |
| hsa-miR-140-5p | hsa-miR-210-5p | hsa-miR-376c-3p | hsa-miR-9-5p |
| hsa-miR-142-3p | hsa-miR-218-5p | hsa-miR-377-5p | hsa-miR-92a-3p |
| hsa-miR-142-5p | hsa-miR-221-3p | hsa-miR-382-5p | hsa-miR-93-5p |
| hsa-miR-143-3p | hsa-miR-222-3p | hsa-miR-422a | hsa-miR-99a-5p |
| hsa-miR-144-3p | hsa-miR-223-3p | hsa-miR-423-5p |  |
| hsa-miR-145-5p | hsa-miR-22-3p | hsa-miR-424-5p |  |
| hsa-miR-146a-5p | hsa-miR-224-3p | hsa-miR-4271 |  |
