# Supplementary material for: Construction Immune Related Feed-Forward Loop Network Reveals Angiotensin II Receptor Blocker as Potential Neuroprotective Drug for Ischemic Stroke
Source: Front Genet. 2022 Mar 28;13:811571. doi: 10.3389/fgene.2022.811571 (PMC8995882; doi:10.3389/fgene.2022.811571)
Supplement: Supplementary file 8 [file Table4.DOCX]

Table S4 Ischemic stroke related TFs.

| Regular TFs | Immune related TFs |
| --- | --- |
| GATA4 | CREB1 |
| HDAC9 | ESR1 |
| HIF1A | FOS |
| NFE2L2 | PPARA |
| NKX2-5 | PPARG |
| NOTCH3 | VDR |
| PARP1 |  |
| PITX2 |  |
| SIRT1 |  |
| SMAD3 |  |
| SMAD4 |  |
| TP53 |  |
| XBP1 |  |
| ZFHX3 |  |
