# Supplementary material for: Construction Immune Related Feed-Forward Loop Network Reveals Angiotensin II Receptor Blocker as Potential Neuroprotective Drug for Ischemic Stroke
Source: Front Genet. 2022 Mar 28;13:811571. doi: 10.3389/fgene.2022.811571 (PMC8995882; doi:10.3389/fgene.2022.811571)
Supplement: Supplementary file 9 [file Table6.DOCX]

**Table S6 A cumulative hypergeometric distribution of miRNA-drug pairs from CFMSN**

| miRNA | durg | no. of miRNA target | no. of drug target | No of common genes | P value |
| --- | --- | --- | --- | --- | --- |
| miR-9-5p | Bezafibrate | 6 | 2 | 1 | 0.192307692307692 |
| miR-9-5p | Doconexent | 6 | 2 | 1 | 0.192307692307692 |
| miR-16-5p | Pomalidomide | 5 | 2 | 1 | 0.128205128205128 |
| miR-20a-5p | Telmisartan | 4 | 2 | 1 | 0.0769230769230769 |
| miR-20a-5p | Bezafibrate | 4 | 2 | 1 | 0.0769230769230769 |
| miR-20a-5p | Doconexent | 4 | 2 | 1 | 0.0769230769230769 |
